# Fabrication of Soft Robotics by Additive Manufacturing: From Materials to Applications

**DOI:** 10.1021/acs.chemrev.4c00749

**Published:** 2025-08-11

**Authors:** Ouriel Bliah, Chidanand Hegde, Joel Ming Rui Tan, Shlomo Magdassi

**Affiliations:** † Casali Center for Applied Chemistry, 26742 Institute of Chemistry, and Center for Nanotechnology and Nanoscience Hebrew University of Jerusalem, Jerusalem 91904, Israel; ‡ School of Materials Science and Engineering, 54761 Nanyang Technological University, Singapore 639798, Singapore; § Campus for Research Excellence and Technological Enterprise (CREATE), Singapore 138602, Singapore

## Abstract

Soft robotics is a rapidly evolving field that leverages the unique properties of compliant, flexible materials to create robots that are capable of complex and adaptive behaviors. Unlike traditional rigid robots, soft robots rely on the properties of soft materials, which enable them to safely interact with humans, manipulate delicate objects, and perform various locomotion processes. This review provides a comprehensive overview of the development process of soft robots by additive manufacturing with a particular focus on the chemical aspects of the materials involved. The types of materials used in soft robotics, highlighting their properties, applications, and the role of their chemical composition in performance, are presented. The review then explores fabrication methods, detailing their chemical underpinnings, advantages, and limitations, followed by presenting common design methods used to optimize soft robots. Finally, the review discusses the diverse applications of soft robots across various domains, including medical, locomotion, manipulation, and wearable devices. By covering every stage of the additive manufactured soft robot, from material selection to application, this review aims to offer a deep and comprehensive understanding of this field.

## Graphical abstract

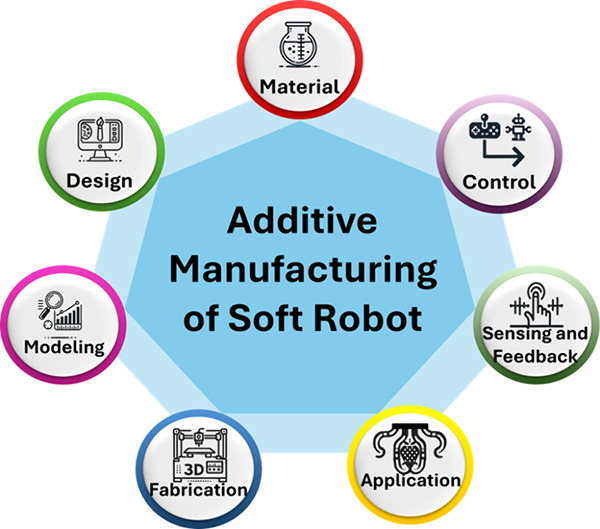

## 1 Introduction

Soft robotics is an emerging field within robotics that diverges from traditional rigid designs by utilizing compliant, flexible materials. These robots rely on the properties of soft materials to allow for a high degree of flexibility and adaptability.^1–3^ Many soft robots are bioinspired, designed to emulate the movement and adaptability of biological organisms, such as the flexibility of octopus arms and elephant trunks, the gentle grasp of human hands, and the locomotion of aquatic, aerial, and terrestrial animals.^4–7^ While rigid robots primarily depend on advanced engineering and programming, the development of soft robots is fundamentally driven by materials science and chemistry, as the selection and design of materials directly dictate their mechanical behavior, actuation mechanisms, and sensing capabilities.^7,8^ Therefore, advancements in these fields have enabled the obtaining of materials with tunable mechanical properties such as flexibility, stretchability, compliance, high impact resistance, and energy dissipation. These customizable properties are critical for developing soft robots to meet specific application needs.^7^

Developing a soft robot involves a comprehensive pipeline that integrates various chemical processes and principles of materials science at every step. This pipeline begins with the planning phase, where specific applications and tasks for the robot are identified. Drawing inspiration from biological systems helps conceptualize the robot’s design and functionality.^9^ Material development considerations are crucial in determining the necessary mechanical properties of the robot for its application and in selecting the appropriate actuation mechanisms, sensing technologies, control, and feedback systems.^10^ Before fabrication, modeling and computational analysis are often performed to predict the robot’s behavior and optimize its design.^11^ Selecting the proper fabrication technique is essential for building the soft robot accurately and efficiently. Among the available options, additive nanufacturing (AM) has become especially influential in the field of soft robotics, offering practically limitless design possibilities that traditional methods like molding or laser cutting cannot. AM enables the use of a wide variety of soft and functional materials, including those that can not be molded or cast, and allows for the fabrication of complex, customized geometries tailored to soft robotic systems’ mechanical and functional needs. In doing so, AM has opened new frontiers in actuator and sensor design, supporting the development of monolithic, multifunctional devices and accelerating innovation in areas such as untethered mobility, wearable interfaces, and bioinspired architectures. Finally, in the last step, the robot is fabricated and tested in real-world applications to ensure that it meets the desired performance criteria.

This Perspective aims to provide a comprehensive overview of the entire process of developing soft robots, with a particular focus on the chemical aspects of the materials involved in the fabrication of soft robots by AM processes. We begin by discussing the various fabrication methods, explaining their chemical underpinnings, advantages, and limitations are discussed. Then, the types of materials used in AM-based soft robotics, highlighting their properties and applications, and emphasizing their chemical composition and how it influences their performance. This is followed by a presentation of the design methods and modeling techniques used to optimize soft robots. Next, we delve into the sensing capabilities of soft robots. Finally, a discussion on the diverse applications of soft robots, demonstrating their impact across various domains, including medical, manipulation, and locomotion, is presented.

While there are numerous reviews on specific aspects of soft robotics, this review aims to provide a comprehensive overview of the entire field, particularly emphasizing the chemical properties and processes that underlie soft robotics. By covering every stage of the soft robot development process, from material selection to application. While conventional fabrication techniques such as molding and casting are occasionally referenced to contextualize material or design choices, this review does not attempt to provide an exhaustive account of non-AM soft robotics. Instead, it selectively includes non-AM examples, where they help illustrate key principles relevant to future AM integration. By delineating this scope, we aim to deliver a deep and focused perspective on how materials and AM technologies are shaping the future of soft robotics.

## 2 Fabrication Technologies for Soft Robotics

As accessibility to 3D printers has grown, there’s been a notable transition in soft robotics from traditional cast fabrication methods to the adoption of diverse 3D printing techniques. This shift is largely due to the increased precision, customization, and design flexibility that 3D printing offers.^12^ Unlike traditional casting, 3D printing allows for the creation of complex geometries, fine-tuned material gradients, and rapid prototyping, which are essential for soft robots that require highly adaptable and intricate designs. Additionally, 3D printing reduces material waste and production time, making it a more efficient and cost-effective option for soft robotic fabrication. Initially, in the early 2010s, cast fabrication dominated soft robot manufacturing.^13^ Yet, in recent years, we have witnessed a shift driven by factors such as material availability and the wide range of required material properties.^14^ It is essential to acknowledge that there is no universal 3D printing solution, each printer’s suitability is heavily contingent upon the materials it can utilize and the specific properties those materials offer for the desired soft robot. While there is not a one-size-fits-all approach, every printer does have its most efficient application niche. Moreover, the selection of the 3D printing method is not solely influenced by material considerations. There’s also been significant exploration into innovative actuation design strategies for soft robots. Initially, soft robots designs were relatively simple, dominated by pneumatic actuation, but the field has since expanded to include more complex architectures and mechanisms that incorporate electrical, magnetic, temperature-responsive, and even chemically responsive actuation mechanisms, which dictate the type of materials to be used in the specific fabrication processes.^12–18^

A wide variety of AM techniques have been adapted for soft robotics, each relying on a different polymerization or solidification mechanism to fabricate soft functional materials. Vat photopolymerization methods such as digital light processing (DLP), masked stereolithography (MSLA), and continuous liquid interface production (CLIP) utilize spatially controlled light to induce radical polymerization, typically involving acrylate- or epoxy-based resins and suitable photoinitiators.^19,20^ Two-photon polymerization (TPP) enables submicroneter resolution through nonlinear optical absorption, using tightly focused femtosecond lasers and photoinitiators with high two-photon absorption cross sections.^21^ In contrast, extrusion-based methods like fused filament fabrication (FFF), commonly known as fused deposition modeling (FDM), and direct ink writing (DIW) rely on thermoplastic flow or viscoelastic extrusion followed by cooling or curing, typically using soft materials such as thermoplastic polyurethane (TPU), silicones, and hydrogels.^22^ Inkjet and aerosol jet printing utilize low-viscosity inks, cured by heat or UV, and are often limited to 2D or pseudo-3D soft structures.^23^ Each printing technique offers trade-offs between resolution, material compatibility, and ease of multimaterial integration, which are critical considerations for functional soft robotic systems. These factors are discussed in detail in section 3.1.

In addition, to overcome the inherent limitations of conventional AM systems, particularly for multimaterial printing, complex kinematics, or unconventional rheologies, researchers have developed a range of customized 3D printing platforms tailored for soft robotics. Innovations include modification of commercial printers, or house-built printers, for example, multinozzle and coaxial extrusion systems,^24,25^ programmable printheads for subvoxel control,^26^ resin vat switching for multimaterial DLP,^27,28^ and integration of magnetic fields^29^ or temperature gradients during printing.^30^ These platforms enable functionalities such as spatial stiffness programming, embedded sensing, actuator, and sensor integration, all of which are essential for untethered and multifunctional soft robotic systems. While these systems offer remarkable design freedom, they often require specialized hardware and software modifications, limiting their accessibility outside research environments.

### 2.1 3D Printing Techniques in View of Soft Robotics Fabrication

In the realm of 3D printing, particularly within the field of soft robotics, the choice of printing technique is deeply intertwined with the materials used,^12^ the intricacy of the designs, and the specific functional requirements of the components.^7^ This chapter compares various 3D printing methods through their effectiveness in soft robotics, evaluating how each technique handles this field demands such as material flexibility, structural details, multimaterial integration, and structural supports. In addition, the effect of printing parameters such as printing speed, availability of custom materials, and the post printing process can affect significantly the soft robot performance are also considered as presented in Figure 1. By analyzing these factors, based on technical specifications from leading 3D printer manufacturers, combined with our perspective tailored to the requirements of soft robotics, this section provides a brief overview of how different 3D printing techniques can be optimized for soft robotics applications, guiding the selection of the most suitable methods for creating individual functional soft robotic systems. In addition, a summary of this section is presented in Table 1.

**Figure 1:**
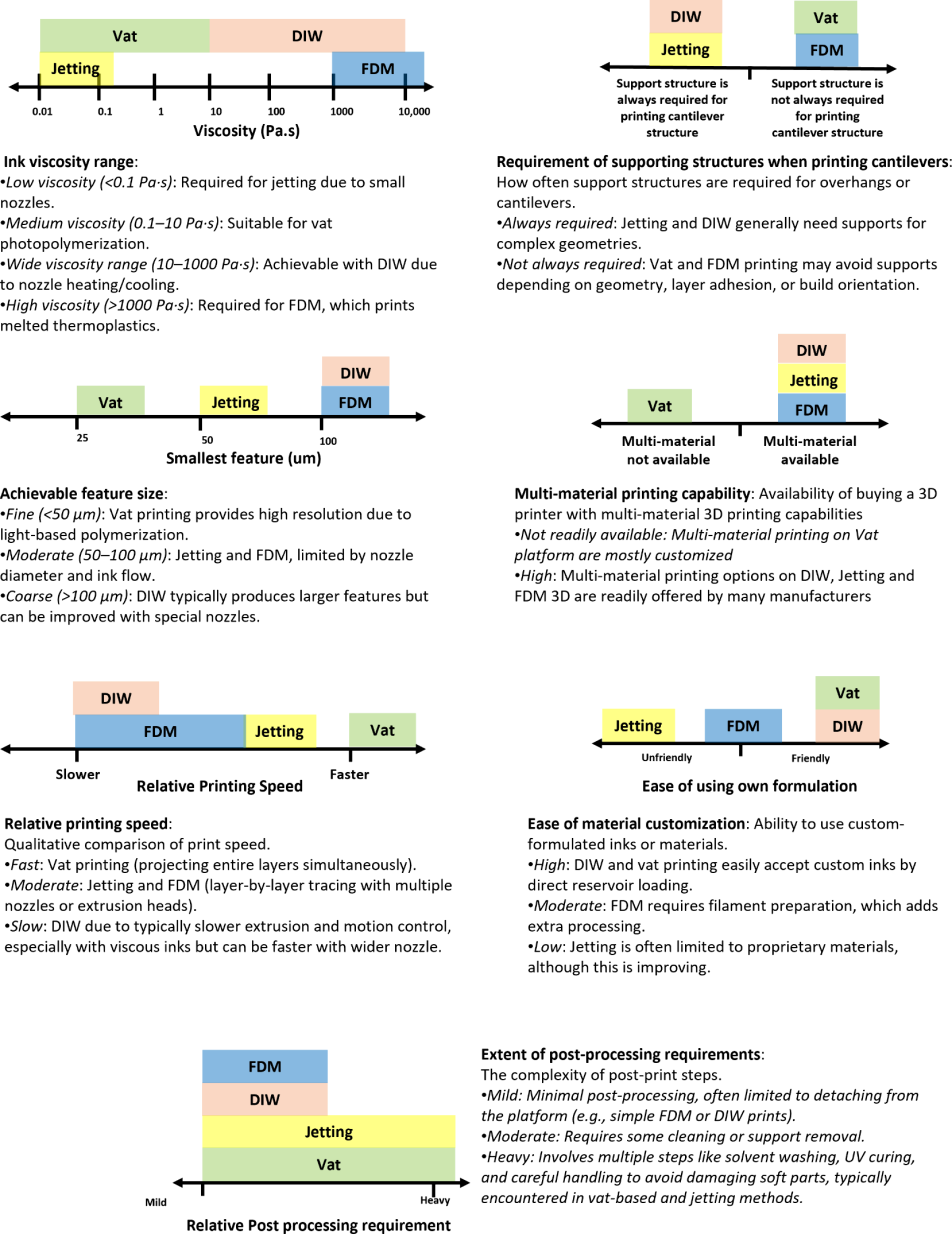
Comparison of the 3D printers based on key criteria relevant for soft robotics: viscosity of ink used, the requirement of supporting structure during printing of cantilever structures, smallest feature size, multimaterial available, relative print speed, ease of material customization, and relative post processing requirements.

**Table 1:** Summary of the 3D Printing technologies, olymerization mechanisms, and key considerations discussed in Section 3.1

Criteria	Jetting	Vat-based (SLA/DLP)	DIW	FFF
Viscosity range	low	up to ∼10 Pa·s; higher viscosities need a heated bath	broad range; must not be too low to avoid dripping	high (melted thermoplastics)
Photopolymer requirement	optional	required (photocurable resins)	optional	no
Support structure needs	high (low-viscosity inks distort shape)	low to moderate; printed upside-down, gravity-assisted	moderate- depends on geometry	low to moderate
Feature resolution	moderate; limited by nozzle and ink channels	high (best resolution with advanced optics; ∼1 μm achievable)	moderate to high; advanced nozzles achieve down to 15 nm	low to moderate; limited by nozzle size
Multimaterial capability	high; mature and fast	low; complex process with washing and ink switching	high; supports multinozzle configurations and material switching	high; multiple extruders enable practical multimaterial use
Printing speed	moderate; multiple nozzles speed up *Y*-axis, but still traces X	high; full layer projection	low to moderate; nozzle traces entire pattern	low to moderate; nozzle tracing like DIW
Postprocessing requirements	high; support removal and UV curing needed	high; solvent washing and UV curing	low; minimal postprocessing	low; minor cleaning
Material development access	low; custom ink testing limited (though improving)	high; direct resin loading enables easy testing	high; highly accessible for formulation and optimization	moderate; requires filament preparation (compounding, extrusion, etc.)
Best use in soft robotics	fine multimaterial parts, when resolution and ink control are needed	intricate, high-resolution parts requiring fast prototyping	custom, complex geometries and soft formulations	robust structural parts or when thermoplastic materials are preferred

The most significant consideration is the material selection, as it directly impacts the printing process. One such aspect is the viscosity, which influences how the material is handled by different 3D printing techniques. In jetting, for instance, the material is expelled through a narrow channel from a small nozzle, usually with a low viscosity.^31^ If the ink’s viscosity is higher, a heating block might be necessary for effective prints. In contrast, common vat-based 3D printing can handle viscosities up to around 10 Pa s, as the polymer can still flow, but higher viscosities may require a heated bath to keep the polymer flowing smoothly.^32^ In DIW 3D printing, the extruder can be either cooled or heated, allowing for a wide range of viscosities. However, the ink’s viscosity should not be too low to avoid dripping issues during printing. FFF printing, on the other hand, employs thermoplastics and operates through melt printing, resulting in generally high viscosity.^33–38^ Another critical aspect in material selection is whether the material is photopolymerizable, as this determines its suitability for methods like SLA and DLP, which rely on light to cure the material, or is polymerized over temperature or time, where then it will suitable for DIW. In the context of soft robotics, these variations in material processability are critical, as they directly affect the performance of the final components, such as soft grippers and actuators, which depend on precise material properties and functionality.

When printing intricate designs, especially those with overhangs or cantilever structures,^39^ it is crucial to include supporting structures to ensure stability during the printing process. In the context of soft robotics, where soft materials are predominantly used, supporting structures are often necessary but may not behave like traditional rigid supports that are easy to remove and leave minimal artifacts on the surface. Instead, these supports can sometimes adhere more strongly or deform the soft material during removal, leading to surface imperfections or compromised functionality. Among the four main printing methods, jetting requires the most supports due to the use of low-viscosity ink,^40^ which leads to the printed pixels losing their intended shape. DIW 3D printing offers versatility in material choice, but supporting structures are often necessary for stable printing. Vat and FFF 3D printing scenarios may not always require supporting structures, depending on factors such as the length and angle of cantilevered sections. Vat 3D printing relies on gravity to prevent collapse, as it prints structures upside down, while FFF 3D printing uses rapid cooling fans to solidify the printed parts. However, the fragility of thin slices and the influence of gravity may still necessitate additional support for successive layers.^41–46^ Therefore, choosing a printing method that minimizes these downsides by reducing the need for extensive supports or enabling easier artifact-free removal is essential to ensure the integrity and performance of the soft robotic components.

For printing features, achieving high resolution is crucial, especially in applications like soft robotics, where precise detail can directly impact functionality. Photon-based printing, such as SLA and DLP, typically achieves the smallest printing features thanks to the advanced optoelectronic and optical adaptors available in the market, therefore making them better candidates for devices that require detailed components.^42,44,47–49^ Jetting, although capable of relatively small features, is not as precise as light-based 3D printing because it requires sizable channels for ink flow and additional parts for ink ejection. Common DIW and FFF processes tend to produce larger printing features due to nozzle limitations. Like jetting, these methods also require sufficiently large channels for material flow, which can restrict the ability to print fine details necessary for certain soft robotic applications. However, in the last years, advancements such as customized nozzles reaching 15 nm resolution for DIW,^50^ and the use of additional optics to achieve 1 μm resolution in vat printing^51^ highlight the ongoing efforts to improve feature resolution in these methods, making them more suitable for this field.

When it comes to multimaterial printing, the ability to integrate different materials within a single print is particularly valuable in applications like soft robotics, where combining materials with varying properties can enhance functionality. While all 3D printing methods have demonstrated multimaterial capabilities, there are notable differences in their practicality and efficiency. Although multimaterial has been shown in customized vat 3D printing in techniques such as ICLIP,^52^ commercial vat 3D printing has not yet fully supported multimaterial printing, often requiring difficult processes such as washing out ink residue from previous prints before immersing the print in another ink for subsequent curing.^28^ This additional step can significantly complicate the process and requires substantial engineering effort to implement in practical applications.^53^ In contrast, jetting, FFF, and DIW offer more mature and accessible multimaterial capabilities, providing a range of options that are better suited for the complex demands of soft robotics.^54–56^

Another consideration in the fabrication of soft robotics is printing speed, particularly because typical soft robots are often large and require rapid prototyping and multiple prototyping to efficiently test designs and performance efficiently. The printing speed varies among 3D printers,^41,42,45,46,57^ DIW and FFF tend to be slower since the nozzle must trace the sliced image, which can be time-consuming. Jetting benefits from having multiple nozzles under the print head, allowing it to cover more distance along the *Y*-axis and speed up printing. However, it still needs to move along the *X*-axis to deposit ink at various locations, according to the sliced file. Vat 3D printing is the fastest option, as it projects the entire 2D sliced image at once, saving significant time. Regarding postprocessing, FFF and DIW 3D printer samples generally require the least amount of time. Vat 3D printing and jetting require more postprocessing, with vat-printed structures needing solvent washing to remove uncured resin and UV postcuring for maximum strength. Similarly, jet-printed structures require jet-washing to remove supporting structures and UV postcuring for optimal structural integrity. In addition, when it comes to multimaterial printing, jetting offers the fastest print time since it does not require cartilage or bath changes like FFF/DIW or DLP, nor does it need to wash previous prints to prevent contamination when switching materials, which is particularly advantageous in the production of complex, multimaterial soft robotic systems.

When developing materials for 3D printing, particularly functional composites, particle dispersion, rheology, and orientation, the factors that influence printability and final device performance. For extrusion-based techniques, such as DIW, poor dispersion (e.g., carbon nanotubes (CNTs), magnetic particles) can lead to clogging, print defects, or mechanical heterogeneity. In vat polymerization methods, a high particle loading may reduce light penetration or inhibit curing efficiency. Moreover, the alignment of anisotropic fillers during the printing process, such as magnetic or conductive particles, can result in directionally dependent (anisotropic) actuation or sensing behavior. While this can be harnessed to enhance functional performance, it also requires careful control over print conditions, field-assisted alignment (e.g., magnetic or electric), and resin formulation. These parameters must be optimized according to the printing platform and the intended robotic function to ensure both structural fidelity and stimulus responsiveness.

Having an accessible platform for printing and material testing is crucial for researchers. Polyjet stands out as the least user-friendly option due to the inability of printer design to allow researchers to test their material. However, this may improve as some companies start offering the ability to print with custom-made inks.^58^ In the field of FFF, even though testing of material requires multiple tools and lengthy processes such as compounding, palletization, and filament pulling to prepare the thermoplastic filaments, these difficulties have been relieved by the easily accessible tools. Given that the advancements in material research is a driver for the evolvement of the field of soft robotics,^7^ the best methods are DIW and vat 3D printing, such are DLP and SLA, since the material is easily inserted to the device and only printing parameter optimizations are required.

## 3 Materials for Soft Robotics

The key to fabricating a soft robotic devices lies in selecting materials that meet the requirements of both the specific mechanical requirements and fabrication method.^7,59,60^ Soft materials are essential in this field because of their unique properties, such as flexibility, stretchability, and energy dissipation, which allow robots to navigate complex environments, absorb impacts, and take on intricate shapes. These materials exhibit viscoelastic behavior, dissipating energy when under load, influencing soft robot design considerations.^3,61,62^ In addition, soft materials form the foundation of components such as actuators and sensors, which enable the robot to move and sense its surroundings.

In this section, soft materials are categorized into two main types: passive and active (i.e., smart) materials, based on their functional role within the robot. Passive materials primarily serve structural purposes without undergoing external stimulus-induced changes, while active materials exhibit responses to external stimuli (e.g., temperature, pH, light, and magnetic fields) to perform actuation or sensing functions. However, this classification is context-dependent, the same material can act as passive or active depending on the intended design and application. For example, a hydrogel may behave as an active material if it undergoes shape change through drying or swelling during operation, but if used solely as a structural scaffold, it would be considered a passive material within the soft robotic system.

### 3.1 Passive Materials

Passive materials form the structural backbone of soft robotic components, providing essential mechanical characteristics, such as elasticity, compliance, and resilience, without being designed to respond actively to external stimuli. These materials enable soft actuators and robotic bodies to perform complex motions when they are actuated. Among soft robotics most widely used passive materials are polyurethane (PU), silicones, and hydrogels. Polyurethane is a versatile polymer known for its excellent mechanical strength, elasticity, and abrasion resistance, making it a common choice for matrix materials in fabricating soft actuators. On the other hand, silicones are highly flexible and easily moldable, allowing them to conform to intricate geometries while maintaining consistent performance even under large deformations. Hydrogels and organogels, while sometimes categorized as smart materials due to their responsiveness under certain conditions, are often employed as passive structural materials in soft robotics. Their unique properties, including high water content and biocompatibility, make them particularly suitable for biomedical applications where safe and soft interactions with tissues are required. In the following section, these common passive materials will be reviewed along with soft robot representatives.

#### 3.1.1 Polyurethane (PU)

Polyurethanes (PU) are versatile segmented copolymers formed by the step-growth polymerization of polyols (soft segment precursors) and diisocyanates (hard segment precursors), typically followed by chain extension with diols or diamines.^63^ The urethane linkage (−NH-CO-O−) is formed via the reaction between the hydroxyl groups of polyols and the isocyanate groups of diisocyanates. The resulting structure consists of alternating soft segments (SS) and hard segments (HS), leading to a microphase-separated morphology.^64^ The soft segments, generally derived from polyether, polyester, or polycarbonate polyols, provide elasticity due to their low glass transition temperature (*T*_g_), while the hard segments, composed of diisocyanate-derived domains, establish physical cross-linking via hydrogen bonding between urethane groups. The mechanical performance and thermal behavior of PU materials strongly depend on the chemical identity, ratio, and molecular weight of soft and hard segment precursors as well as the extent of phase separation between these domains.

Thermoplastic polyurethanes (TPUs) are a class of PUs that maintain melt processability due to reversible physical cross-links and microphase separation. The elastomeric characteristics of these compounds stem from the copolymer interface formed between the soft and hard segments of the polymer. The hard-segment urethane domains act as cross-linkers for the soft-segment amorphous polyester (or polyether) domains. The separation of these domains arises from the inherent incompatibility and immiscibility of the soft segments (characterized by low melting points and nonpolar nature) with the hard segments (characterized by high melting points). The covalent coupling between the hard and soft segments inhibits plastic flow within the polymer chains, resulting in elastomeric resilience. Mechanical deformation of these compounds causes certain portions of the stressed soft segment to uncoil, leading to the alignment of the hard segments along the direction of the stress. In conjunction with strong hydrogen bonding, this realignment contributes to high tensile strength, tear resistance, and good elongation properties in the material. In recent years, the development of better extruder design of FFF printers and the advancement in formulation and manufacturing of TPU filaments have equipped the community with a highly accessible tool for 3D printing of soft robotics design.

Currently, varying shore hardness of TPU filament is available off-the-shelf, and various works on 3D printing of soft grippers, joints, and interface design have been reported as well,^65,66^ key examples are shown in Figure 2. Reported works include mechanical stiffness augmentation of a 3D printed soft prosthetic finger,^67^ high-force soft printable pneumatics for soft robotics applications,^68^ and direct printed soft gripper with adjustable stiffness.^69^ Stano et al. reported on a 3D printed monolithic bending PneuNets (MBPs), a class of soft and monolithic gripper.^70^ They used a dual-extruder 3D printer (Ultimaker 3) to 3D print these actuators. The materials used were two types of TPU: TPU 95A for the rigid portions and TPU 80A LF for the extensible parts of the actuator. Their scientific breakthrough lies in developing a novel, airtight embedded air connector (EAC) integrated directly into the 3D printed structure, overcoming traditional air leakage issues. They optimized the design and printing parameters, demonstrating significant improvements in the actuator’s bending performance by reducing the wall thickness while maintaining air tightness, thus advancing the field of soft robotics fabricated using FFF. Tawk et al. developed a 3D printed modular soft gripper integrated with metamaterials designed for conformal gripping.^71^ They utilized a commercially available TPU (NinjaFlex) and fabricated the gripper using a FFF 3D printer (FlashForge Inventor). The soft gripper’s design significantly enhances its conformability, reduces out-of-plane deformations, and improves the stability and effectiveness of its grasping capabilities. This innovation allows the gripper to handle a wide variety of objects with increased precision and reliability, making it a strong candidate for universal grasping applications. Zhai et al. reported 3D printed monolithic soft robotic^72^ (Figure 2A) devices with embedded fluidic control circuits, including an autonomous gripper designed to perform gripping and releasing tasks autonomously. They printed their soft gripper on a FFF printer (Raise3D E2) with a TPU filament. Their uniqueness lies in developing a method to create complex, airtight, and high-performance soft pneumatic devices using FFF, particularly through the innovative application of Eulerian path printing techniques. This allowed them to achieve seamless, continuous prints without requiring manual postprocessing or assembly. The unique soft robotic mechanism that they developed involves integrating fluidic control circuits directly into the 3D printed structure, enabling electronics-free autonomous operation, which significantly advances the capabilities and accessibility of soft robotics.

**Figure 2:**
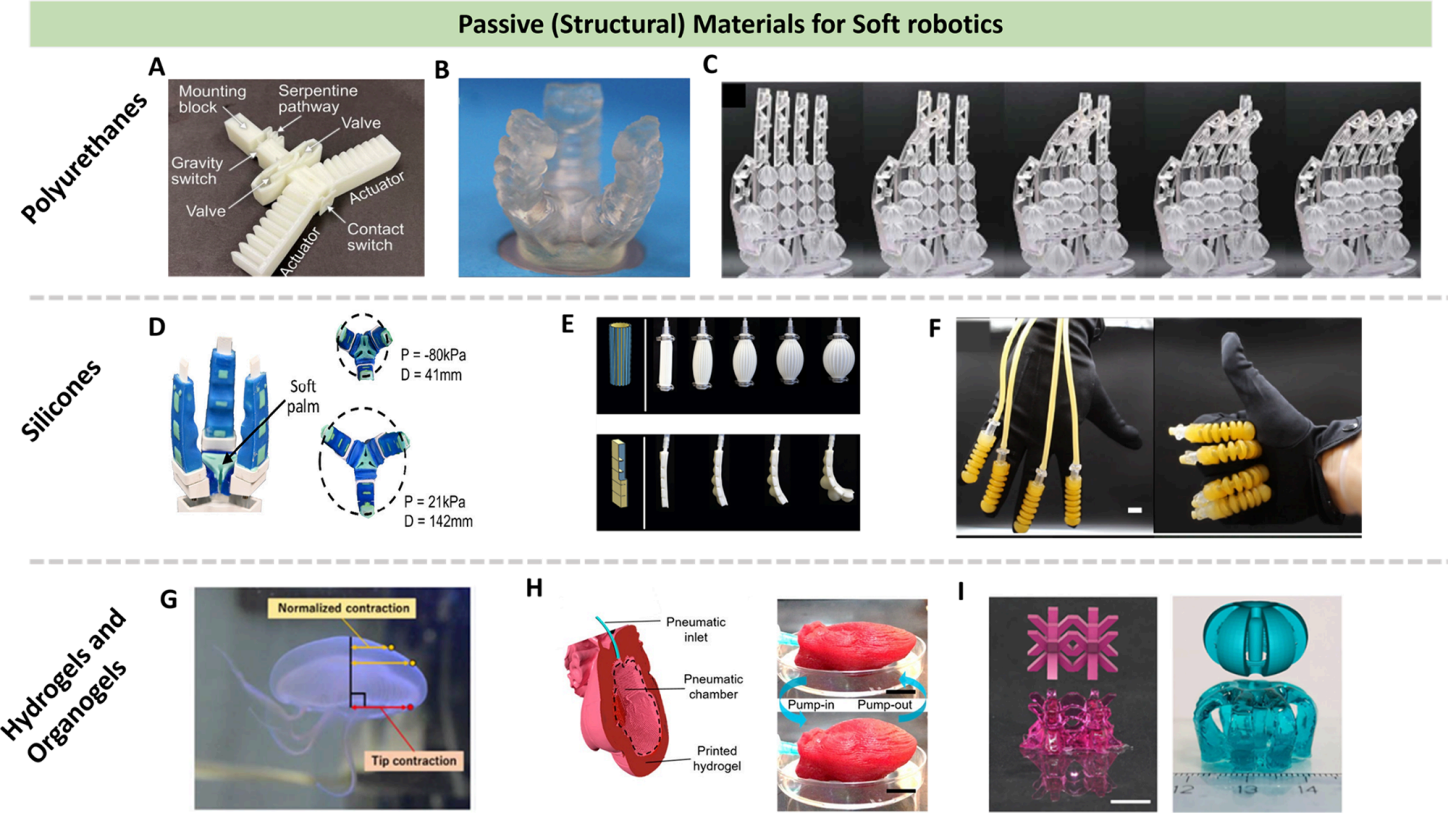
Passive materials for soft robots discussed through section 4.1: Polyurethanes. (A) TPU-based monolithic soft robotic devices with embedded fluidic control circuits. Reproduced with permission from ref 72. Copyright 2023 AAAS. (B) PUA-based highly stretchable pneumatic actuators. Reproduced with permission from ref 32. Copyright 2017 Wiley-VCH. (C) PUA-based biomimetic artificial muscle. Reproduced with permission from ref 79. Copyright 2022 AAAS. Silicones. (D) Freeform liquid 3D printing of soft gripper. Reproduced with permission from ref 91. Copyright 2021 American Chemical Society. (E) 3D printed programmable bioinspired architecture soft actuators. Reproduced with permission from ref 117. Copyright 2018 Springer Nature under the Creative Commons Attribution 4.0 International License (CC BY 4.0). (F) Printed tough silicone-based actuators with double silicone networks. Reproduced with permission from ref 105. Copyright 2021 Springer Nature under the Creative Commons Attribution 4.0 International License (CC BY 4.0). Hydrogels and Organogels. (G) 3D printed actuator for jellyfish-like locomotion. Reproduced with permission from ref 112. Copyright 2021 The Electrochemical Society under the Creative Commons Attribution 4.0 International License (CC BY 4.0). (H) DIW 3D printed hydrogel-based biomimetic soft robotics. Reproduced with permission from ref 114. Copyright 2021 American Chemical Society. (I) Formulation of self-healing hydrogel. Reproduced with permission rom ref 110. Copyright 2021 Published by Springer Nature under the Creative Commons Attribution 4.0 International License (CC BY 4.0).

Urethane acrylates (UAs) are polyurethane-derived oligomers or monomers functionalized with (meth)­acrylate groups, enabling radical polymerization through photoinitiated or thermally initiated mechanisms.^73^ They serve as key components in vat photopolymerization (VP) resins due to their ability to form cross-linked networks upon curing. UAs can be synthesized in a broad range of molecular weights, resulting in formulations ranging from low-viscosity monomeric liquids to highly viscous oligomeric pastes. The structure–property relationship of the resulting polyurethane acrylate (PUA) networks is highly tunable, influenced by the chemical nature of the UA backbone, the functionality and concentration of reactive diluents, and the choice of cross-linking density. Like TPU, PUA-based materials retain elastomeric behavior originating from flexible urethane linkages within the backbone. However, unlike TPU, which relies on phase-separated soft and hard domains for elasticity, PUA achieves elasticity primarily through covalent cross-linking and network design. The incorporation of low-viscosity reactive diluents, such as hydroxyethyl acrylate or isobornyl acrylate, significantly affects the mechanical properties of the final PUA, affecting both tensile strength and elongation at break by altering the network’s cross-link density and segmental mobility.

Currently, the products, which offer stretchable UA-based UV 3D printable inks on off-the-shelf vat printers and give soft and flexible 3D prints, are available. The work includes synthesis of PUA for the formulation of highly stretchable ink,^74^ formulation of compressible PUA ink by introduction of pores,^75^ and design and printing of bellow actuators for locomotion.^76,77^ Patel et al. reported on a formulation of a highly stretchable and UV-curable ink^32^ (Figure 2B) specifically designed for DLP 3D printing. The formulation consists of epoxy aliphatic acrylate (EAA) and aliphatic urethane diacrylate (AUD). The uniqueness lies in the stretchability of up to 1100%, which is more than five times the elongation at break of commercial UV-curable elastomers. This innovative formulation allows them to directly 3D print complex, highly deformable structures, including soft actuators and gripper. Ge et al. shared a work on customization of a DLP 3D printer^78^ capable of fabricating soft pneumatic actuators of various sizes with exceptional speed and precision. The printing process utilizes projection microstereolithography, enabling the creation of complex structures with high accuracy. Additionally, they designed a soft pneumatic gripper featuring three micropneumatic actuators, each with 0.4 mm wide square air channels and 0.2 mm thick chamber walls. Their gripper was integrally printed in less than 30 minutes, demonstrating the efficiency and effectiveness. Pascali et al. shared a work on 3D printed biomimetic artificial muscles^79^ (Figure 2C) called GeometRy-based Actuators that Contract and Elongate (GRACE). These artificial muscles are designed as soft actuators that can both contract and elongate, mimicking natural muscle movements. The primary material used for these actuators was UV resin on a DLP 3D printer. The scientific breakthrough of this work lies in the development of a monolithic pleated membrane design that enables both contraction and elongation without the need for additional strain-limiting components or end-caps, significantly simplifying the fabrication process. Zhang et al. 3D printed miniature pneumatic actuators,^80^ focusing on creating high-resolution, multimaterial soft actuator. The UV ink based on TangoPlus mixed with epoxy aliphatic acrylate was used. The multimaterial UV 3D printer enables direct printing of multimaterial structures with anisotropic properties, such as a soft gripper with helical actuation, enhancing the functionality and versatility of the printed devices.

#### 3.1.2 Silicones

Silicone, also known as polysiloxane, widely applied in various fields today, owing to its exceptional thermal stability, biocompatibility, diverse softness levels, and mechanical robustness.^72^ The physical and mechanical attributes of the resultant polymer can be tailored based on the reactive functional groups in its molecular structure, such as hydride, alkene, and alcohol moieties. Typically, silicone curing entails cross-linking reactions, with minimal involvement of propagation steps, as fully described in the section that makes silicone-based polymers predominantly thermoset in nature. The thermoplastic silicone formulations are primarily block copolymers and linear silicone polymers.^81,82^ Polydimethylsiloxane (PDMS) is the most commonly used form of polysiloxane and has played a central role in the development of soft actuators, such as in the field of microfluidics and soft lithography due to its optical clarity, flexibility, and ease of processing.^83,84^ Direct 3D silicone based printing includes rapid liquid printing (gel-bath-based) of silicon structure complex and soft robotics,^85^ formulated silicone-based ink for 3D printed programmable bioinspired architectures,^86^ rapid thermal curing of direct complex silicone printing outside gel-bath,^87^ artificial muscle and locomotions,^88–90^ and freeform 3D printing of a functional pneumatic gripper.^91–93^ 3D printed silicone based soft robotics is presented in Figure 2, and detailed below.

Li et al. reported on the development of custom-built multimaterial embedded 3D printing (EMB3D) for fabricating multifunctional components in soft robotics.^94^ These components included complex silicone-based structures such as a sensorized compliant fishtail and a pneumatic humanoid hand. They utilized a silicone-based material system involving a platinum catalyst ink and a silicone oil matrix, allowing for seamless integration and robust mechanical properties. Wehner et al. reported on the development and 3D printing of silicone elastomer soft robots,^95^ comparing their performance to traditionally molded counterparts. They designed and 3D printed soft robotic structures, including a four-channel tentacle, a pneu-net actuator, and a soft quadrupedal robot, all using a custom-built 3D printer equipped with a specialized extrusion mechanism. The material used was Dragon Skin 10 Very Fast, a platinum-cured silicone, which was modified with a viscosifying agent to enhance the print fidelity. Their study also identified a potential “sewing thread effect”, where layer-by-layer printing reinforces the structure, enhancing its strength and reliability.

Calais et al. reported on the development of a freeform liquid 3D printing (FL-3DP) technique for fabricating functional components for soft robotics^91^ (Figure 2D). They designed and 3D printed customized functional sleeves and multimaterial pneumatic components, such as soft grippers, using a nanoclay-modified support bath and room-temperature vulcanized (RTV) silicone-based inks. The custom-built FL-3DP system enabled the integration of complex multimaterial structures with superior geometric freedom and robust material interfaces compared to traditional casting methods. Their unique contribution includes the precise control of ink and support bath interactions, allowing for the seamless integration of materials with different mechanical properties, which significantly enhances the performance and durability of soft robotic devices.

Historically, casting has served as the primary method for fabricating silicone structures. However, 3D printing techniques such as DIW, vat, and inkjet for silicone parts have been gaining attention. Among these, DIW stands out as a prominent method, particularly for its ability to yield silicone structures with properties similar to those obtained through traditional casting processes. Leveraging on UV radical acrylate and/or thiol–ene cross-linking chemistries, formulations of silicones and polysiloxane-based inks have been reported for printing of a silicone-based 3D model with various mechanical properties. However, it is to be noted that silicone printing via UV radical polymerization requires inks with good flow properties, often resulting in shorter oligomer chains and subsequently compromised mechanical performance as compared to their thermal silicone counterparts. The majority of the current reports on UV curable silicones focuses on formulations to tune the mechanical properties of the UV cured silicone material,^96–103^ formulated silicone-based ink for 3D printed programmable, self-healing properties,^104^ while reports on direct UV 3D printed soft robotics are not common.

Schaffner et al. reported on 3D printed soft actuators with programmable bioinspired architectures^86^ (Figure 2E). These actuators were designed to mimic complex motions such as twisting, bending, and contracting. They used a custom-formulated silicone ink named “Silink”, which has tunable elasticity and is based on photocurable silicone materials. While using a printer with multimaterial DIW platform, they printed bioinspired fiber architectures, allowing for intricate motion similar to natural muscular hydrostats, such as elephant trunks and octopus arms.

Wallin et al. reported on the development and 3D printing of tough silicone double networks (SilDNs)^105^ (Figure 2F) designed for soft robotic applications. These networks were created to achieve a combination of low elastic modulus and high toughness, making them suitable for simulating the mechanical properties of soft tissues and for use in advanced robotics. They utilized a custom silicone formulation that combined a thiol–ene photocurable network with a condensation-cured silicone network. The 3D printing was carried out using an SLA printer, which allowed for high-resolution fabrication of complex geometries.

#### 3.1.3 Hydrogels and Organogels

Hydrogels are 3D networks formed from hydrophilic polymers that can absorb and retain substantial amounts of water without dissolving. Their ability to maintain a defined shape in aqueous environments stems from a combination of covalent and noncovalent interactions. Chemical cross-linking, involving covalent bonds between polymer chains, establishes a permanent and stable network structure. In contrast, physical cross-linking, mediated by noncovalent interactions such as hydrogen bonding, hydrophobic interactions, ionic interactions, and crystallite formation, provides reversible and dynamic connectivity.^106^ In polyelectrolyte hydrogels, electrostatic interactions between charged groups along the polymer chains play a significant role in both swelling behavior and mechanical integrity.^107^ Additionally, physical entanglements, especially in systems with high molecular weight polymers, act as transient cross-links. However, the high water content in hydrogels often leads to increased chain mobility and reduced effective cross-link density, typically resulting in lower tensile strength compared to other soft solids. This intricate interplay of interactions confers hydrogels with tunable mechanical and functional properties, making them highly adaptable materials, which is a preferable property for soft robotics. In addition, bioprinting techniques, particularly extrusion-based methods, have also been employed to fabricate hydrogel-based soft robotic structures with complex architectures, enabling spatial control of composition and potential integration with biological systems.^106^

Organogels share many structural similarities with hydrogels but are formed by incorporating an organic liquid instead of water as the dispersion medium. These gels consist of a three-dimensional network of polymers or low molecular weight gelators that immobilize organic solvents through physical or chemical interactions.^108^ In the context of soft robotics, organogels offer lower volatility, broad chemical compatibility, and often enhanced mechanical strength compared to hydrogels.^109^ Their tunable rheological and viscoelastic properties and solvent-specific responsiveness make organogels attractive candidates for actuators and sensors that require stability in nonaqueous media or environments where water is incompatible. However, it is worth noting that organogels are still not widely used in soft robotics fabricated by AM compared to hydrogels, PUA, and silicones.

Hydrogels and organogels represent a versatile material class that can be utilized as either passive or active components in soft robotics, depending on the functional design of the system. For instance, hydrogels function as active materials when used as stimuli-responsive actuators. However, when incorporated purely as compliant scaffolds or structural matrices without exploiting their stimulus-responsiveness or for intrinsic self-healing properties,^110,111^ they are considered passive. In the context of this section, hydrogels are discussed as passive structural materials in soft robots^112–114^ and shown in Figure 2 and detailed below. For instance, Takishima et al. reported the development of a fully 3D-printed hydrogel actuator designed for jellyfish-mimicking soft robots^112^ (Figure 2G). The actuator composed of three main parts, Connector, Box, and Base, was fabricated using a light-scanning-type 3D gel printer, which enables precise control over the elastic properties and bending behavior of the actuator. The hydrogel used in the actuator was a high-strength, particle double-network (P-DN) gel, known for its high water content and 3D printability. The study demonstrated that the 3D-printed actuator’s motion closely resembles that of a moon jellyfish, indicating its potential applicability in jellyfish-mimicking robots for underwater exploration. Cheng et al. reported on the DIW 3D printing of hydrogels into biomimetic soft robots^114^ (Figure 2H). These soft robots were designed to perform complex, nature-inspired motions, such as the fluidic actuation of an artificial tentacle, the rhythmic beating of a bioengineered robotic heart, and the phototropic movement of an artificial tendril. They used alginate as a rheological modifier to maintain the desired properties of the host hydrogels, while enhancing their mechanical toughness.

Ge et al. reported on the development of a multimaterial 3D printing^113^ approach for fabricating highly stretchable hydrogel structures bonded with diverse UV-curable polymers. They used a custom-built DLP-based 3D printer to create complex hybrid structures composed of an acrylamide-PEGDA (AP) hydrogel and other polymers like elastomers, rigid polymers, and shape memory polymers (SMP). The AP hydrogel, known for its high water content and stretchability, was made UV-curable using water-soluble TPO nanoparticles, enabling the formation of strong covalent bonds at the hydrogel–polymer interface.

Naranjo et al. reported on the development of an autonomous self-healing hydrogel for soft robotics applications.^111^ They 3D printed a pneumatic artificial muscle (PAM) using a hydrogel-based material named SHAP, which exhibits autonomous self-healing properties without the need for external stimuli. The material used was a hydrogel based on [2-(acryloyloxy)­ethyl]­trimethylammonium chloride (AETA), further enhanced with few-layer graphene (FLG) to improve the mechanical properties. The utilized 3D printer was not specified as a commercial product but rather as a custom setup designed for the fabrication process. Caprioli et al. reported on the development of a self-healing hydrogel suitable for 3D printing using DLP 3D printer^110^ (Figure 2I). The hydrogel, composed of a semiinterpenetrated polymeric network (semi-IPN), enables autonomous self-repair at room temperature without external stimuli. The material consists of poly­(vinyl alcohol) (PVA), acrylic acid (AAc), and poly­(ethylene glycol) diacrylate (PEGDA), with the self-healing mechanism driven by hydrogen bonding. The use of commercially available materials and a standard DLP printer allows for the fabrication of complex 3D structures, with the hydrogel recovering 72% of its original strength after 12 h.

Examples of organogels include a work by Xin et al., who developed a 3D-printed electrohydrodynamic pump using a DLP-fabricated antiswelling organohydrogel composed of a PUA-based organogel and an acrylamide–PEGDA hydrogel. The printed material exhibited Young’s modulus of 0.33 MPa, 300% stretchability, and <10% swelling, enabling robust mechanical performance under bending and twisting. The soft pump achieved a record-high pressure of 90.2 kPa and 800 mL/min flow rate, powering various soft robotic systems, including a gripper, a climbing worm, and a swimming squid. This work highlights organohydrogels as promising actuator enablers for untethered, high-performance soft robots.^115^ Moreover, Li et al. developed a family of silicone-based organogel inks for direct ink writing (DIW), enabling the fabrication of multimaterial, biocompatible soft structures with tunable mechanical properties. The inks, composed of photo-cross-linkable silicones, silicone oil, and fumed silica nanoparticles, span elastic moduli from ∼13 to ∼530 kPa. The authors demonstrate graded and patterned 3D-printed structures capable of controlled buckling and nonlinear.^116^

#### 3.1.4 Comparison of Passive Materials for Soft Robotics

The selection of passive materials is critical in the design of soft robotic systems, as it directly influences actuation modes, mechanical performance, fabrication methods, and functional lifespan, where each material offers distinct advantages and limitations depending on the targeted application. In this subsection, a comparison between the above-mentioned materials is made, along with a summary table that includes smart materials, as presented below (Table 2).

**Table 2:** Comparison of Smart Material Classes for Soft Robotics, Including Key Material Examples, Printing Methods, Advantages, and Limitations

Stimulus type	Material examples	Printing methods	Advantages	Limitations
Electrical	DEA, IPMC, electroactive hydrogel	DIW, SLA, multimaterial FFF	fast response, high spatial resolution, reversible actuation, sensing integration	high voltage (DEA), limited durability, environmental sensitivity (IPMC)
Magnetic	NdFeB composites, Fe_3_O_4_, CIP in TPR or silicone matrix	FFF, DLP, extrusion with magnetic field	untethered, remote actuation, shape programmability, multimodal motion	low force output, fatigue under cycling, complex alignment during printing
Thermal	SMPs, PCL and PUAs derviatives with CNT or carbon black	DLP, FFF, DIW, SLA	large deformation, reprogrammable shapes, self-healing integration	slow response, often one-way actuation, needs external heating
Photo	rGO-PNH hydrogels, Gold nanorod LCEs, Azobenzene-PUA	DIW, SLA, Stereolithography	contactless, spatially targeted, dual-wavelength reconfiguration	low mechanical strength, slower response, light alignment requirements

Polyurethanes (PU and TPU) are widely favored for their tunable mechanical properties, ranging from highly elastic soft segments to rigid, crystalline hard segments. Its inherent toughness, high tear resistance, and resilience to mechanical deformation make it suitable for components exposed to cyclic loading such as grippers and joints. TPU, in particular, is compatible with FFF methods, enabling direct 3D printing of soft robotic components. However, the drawbacks of polyurethane include moderate biocompatibility (compared to silicones) and potential degradation when exposed to moisture, UV, or extreme temperatures over extended periods.

On the other hand, silicone (polysiloxane-based materials) is known for its excellent thermal stability, chemical inertness, and high biocompatibility, making it ideal for bioinspired actuators, medical soft robots, and wearable devices, as will be shown in section 7. Its highly elastic nature and customizable mechanical properties (through the choice of cross-linking chemistry and filler modification) allow for the creation of structures mimicking soft tissues. Additionally, silicones are compatible with direct ink writing (DIW) and embedded printing techniques. However, silicone suffers from relatively low tensile strength and tear resistance compared to polyurethane and typically requires postcuring processes. Moreover, direct UV-curable silicone printing still faces challenges in achieving high mechanical performance due to the low molecular weight of photocurable precursors.

Hydrogels or organogels stand out due to their high liquid content, biocompatibility, and potential for stimuli-responsiveness, making them particularly suitable for biohybrid soft robots, actuators, and applications requiring reversible deformation. They are ideal for soft robots mimicking living organisms, especially in underwater environments, for hydrogels. However, they inherently have lower tensile strength relative to silicone and PU/TPU and limited mechanical durability due to their low intermolecular interactions and high porosity. Their mechanical weakness often restricts their use to applications in which high loads are not expected. Furthermore, maintaining the long-term structural integrity under drying is a significant challenge.

### 3.2 Smart Soft Materials

Smart materials are a class of active materials capable of changing their physical or chemical characteristics in response to external stimuli such as moisture, pH, light, temperature, or magnetic fields. In soft robotics, these materials are essential for enabling components that can adapt, actuate, or recover autonomously, greatly expanding the functional capabilities of soft devices. The integration of smart materials into additive manufacturing has given rise to the emerging field of 4D printing, where time-dependent or stimulus-induced transformations are incorporated as additional design dimension. Common soft smart materials are composites, active self-healing materials, and liquid crystal elastomers (LCEs). Composites are typically formed by embedding functional fillers, such as carbon nanotubes (CNTs), conductive polymers, magnetically responsive particles, conductive nanoparticles, and shape-memory additives, into a passive soft matrix, granting the material a controllable response to external stimuli.^118,119^ Active self-healing materials offer the ability to autonomously repair structural damage when activated by stimuli, often through mechanisms involving dynamic bonds or reversible polymer networks,^120,121^ and LCEs combine the elasticity of polymer networks with the anisotropic properties of liquid crystals, allowing for reversible and programmable shape changes.^122^ In soft robotics, smart materials are commonly classified based on the type of stimulus that drives their actuation, rather than the intrinsic material properties alone. This chapter focuses on four main types: electrically, magnetically, and thermally actuated materials and photoactuated materials, each with representative soft robot examples presented below.

#### 3.2.1 Electrically Actuated Robotics

Electrically responsive materials (ERM) exhibit a remarkable ability to undergo shape changes when subjected to an external bias. This transformative property enables the realization of directional actuation, a feat accomplished through control of the shape alteration, and utilized for printing actuators for soft robotics, as shown in Figure 3. ERM can be dielectric elastomer actuators (DEA),^123^ ionic polymer–metal composite (IPMC),^124^ or polymer–CNT composites.^125^

**Figure 3:**
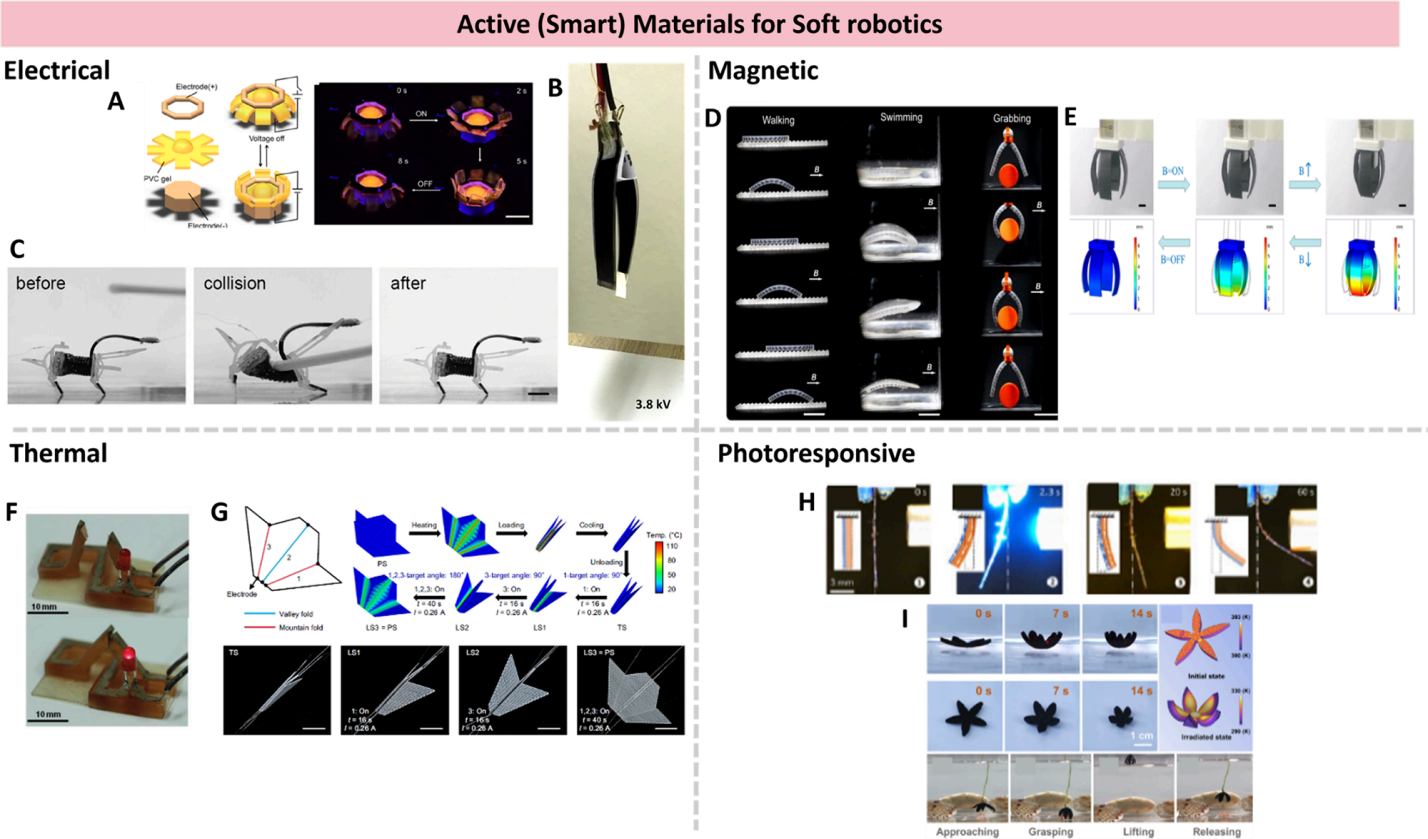
Active materials for soft robots are discussed in section 4.2. Electrical (A) PVC based electrical responsive actuator. Adapted with permission from ref 132. Copyright 2021 American Chemical Society. (B) FFF printed soft DEA. reproduced with permission from reference 137. Copyright 2023 Wiley-VCH under the Creative Commons Attribution 4.0 International License (CC BY 4.0). (C) 3D printed DEA-based insect-scale ultrafast soft robot. Adapted with permission from ref 138. Copyright 2023 American Chemical Society. Magnetic. (D) Shape programmable actuators for biomimetic inspired locomotion. Reproduced with permission from reference 143 Copyright 2020 Elsevier. (E) Magnetic field-induced deformation for grasping applications. Reproduced with permission from ref 144. Copyright 2021 American Chemical Society. Thermal. (F) Fabrication of shape memory-based electrical devices. Reproduced with permission from ref 152. Copyright 2015 Wiley-VCH. (G) Electrothermally controlled origami fabricated by 4D printing of continuous fiber-reinforced composites. Reproduced with permission from ref 153. Copyright 2024 Springer Nature under the Creative Commons Attribution 4.0 International License (CC BY 4.0). Photoresponsive. (H) Polyurethane-urea based elastomer bidirectional actuation driven by photochemical and photothermal coupling mechanism. Reproduced with permission from ref 168. Copyright 2024 American Chemical Society Creative Commons Attribution 4.0 International License (CC BY 4.0). (I) Fast near-infrared light response actuation. Reproduced with permission from ref 173. Copyright 2024 American Chemical Society.

The IPMC comprises an ionic polymer immersed in an ionic solvent and ensconced between two electrodes. When subjected to external bias, the cation/ion species with a distinctive size difference migrate toward opposing electrode poles, inducing a shift in volume occupancy proximate to the electrodes. The differential response between smaller and larger molecules results in a shrinkage on one end and an expansion on the other. This movement in molecules culminates in the bending of the IPMC stack toward the side experiencing shrinkage, thus effectuating motion or actuation. There are works focusing on the formulation of 3D printable IPMC for conductivity^126–128^ stretchability,^129^ as well as direct 3D printed IPMC for manipulation and locomotion.^130,131^ For example, Han et al. reported on the development of a soft robotic system utilizing a 3D-printed electroactive hydrogel (EAH) for manipulation and locomotion applications.^131^ They employed a projection microstereolithography (PμSL) technique to fabricate complex 3D EAH structures that exhibit large deformations in response to electric fields. The hydrogel was composed of acrylic acid (AA) and poly­(ethylene glycol) diacrylate (PEGDA 700), cross-linked with a photoinitiator. The study demonstrated the hydrogel gripper’s ability to perform tasks such as gripping and transporting objects as well as bidirectional locomotion. The precise dimensional control offered by the PμSL technique allowed for the creation of actuators with varying thicknesses, enabling tailored actuation speeds and complex deformations. In addition, Wang et al. reported on the development of electrically responsive soft actuators using 3D-printed polyvinyl chloride (PVC) gel^132^ (Figure 3A). They fabricated a jellyfish-like actuator from PVC ink, which can achieve a 130° bending in less than 5 s under an electric field. The PVC gel, composed of a PVC network and a plasticizer, responds to an electric field by creeping toward the anode due to electrical Maxwell stress and electrowetting effects. A custom DIW setup was utilized for the 3D printing process, allowing for the creation of actuators with varying geometries and stiffness gradients and enabling undulatory motion.

In contrast, DEA stacks feature a soft dielectric layer sandwiched between two electrodes. The application of external bias instigates Maxwell stress between the electrodes, exerting pressure on the center dielectric layer. Consequently, the surface area parallel to the electrodes undergoes expansion, while the perpendicular surface contracts. This modulated motion serves as the foundation for various configurations, including rolling, conical, and folded geometries. These configurations translate planar-specific deformation into deliberate directional actuation. The wide variety of work includes 3D printed multilayer dielectric for haptic^133^ and multimaterial DEAs.^134,135^ Chortos et al. reported on the development of 3D-printed interdigitated DEAs for soft robotics applications.^136^ The team utilized a DIW method to fabricate vertical interdigitated electrodes, which were subsequently encapsulated in a self-healing dielectric matrix made of plasticized, chemically cross-linked polyurethane acrylate. These DEAs demonstrated in-plane contractile actuation with strains of up to 9% and a breakdown field of approximately 25 V/μm. The versatility of the DIW technique allowed for the creation of complex geometries, including prestrain-free rotational actuators and multivoxel DEA with orthogonal actuation directions. Raguž et al. reported on the development of a soft dielectric actuator produced entirely by multimaterial FFF 3D printing^137^ (Figure 3B). They fabricated the actuator using a combination of commercially available TPU filaments for the dielectric membrane and electrically conductive filaments for the electrodes. The study explored the influence of membrane thickness and electrode printing direction on the actuator’s performance, optimizing the fabrication process to achieve a maximum displacement of 42%. The utilized 3D printer was a dual-extruder FFF machine, and the resulting actuator was showcased in a soft dielectric gripper, demonstrating the potential of fully 3D printed soft actuators for practical applications. In another work, Zhu et al. reported on the development of a high-frequency DEA designed for insect-scale ultrafast soft robotic^138^ (Figure 3C). They employed a 3D printing technique to fabricate the actuator, which exhibited rapid actuation speeds with a resonant frequency of up to 1000 Hz. The DEA was composed of a highly stretchable dielectric elastomer layer sandwiched between two compliant electrodes, allowing for large deformations under electric fields. The material used for the dielectric elastomer was a UV-curable silicone elastomer optimized for high-frequency actuation. The utilized 3D printer was a custom setup specifically designed to achieve the fine resolution needed for fabricating such small-scale actuators. Haghiashtiani et al. reported on the development of 3D-printed electrically driven soft actuators for soft robotics applications.^139^ They fabricated a unimorph DEA using a DIW method to print a layered structure composed of a silicone-based dielectric layer and ionic hydrogel electrodes. The DEAs demonstrated significant bending motion in response to applied electrical stimuli, achieving a maximum vertical tip displacement of 9.78 ± 2.52 mm at 5.44 kV. The materials used, including a UV-curable silicone matrix enhanced with barium titanate nanoparticles, were optimized for printability and performance.

#### 3.2.2 Magnetically Actuated Robotics

Magnetically responsive materials are those that change their shapes in response to a magnetic field. As with other actuators, the motion should stop once the external stimulus is removed. In magnetically responsive materials for soft robotics, soft magnetic materials are preferred over superparamagnetic ones because soft magnetic materials retain magnetization only when the magnetic field is applied. In contrast, superparamagnetic materials continue to exhibit magnetic behavior for a short time after the external field is removed, which can cause unwanted residual motion.^140^ Soft magnetic materials are characterized by high magnetic susceptibility and saturation magnetization but with relatively low remanence and coercivity. Hence, soft-magnetic materials (e.g., iron oxide) are strongly attracted to a magnet and easy to magnetize, but at the same time, they are also easily demagnetized by a relatively weak magnetic field. But by incorporating the powder of soft magnetic materials into a soft polymeric matrix, it is possible to achieve soft robotics actuation by applying an external magnetic field to the composite as shown in the examples in Figure 3. Magnetic smart materials have been used for 3D printing of biomimetic applications and locomotion via programmable magnetization^141–146^ and magnetic induced grasping motion.^147,148^

Qi et al. reported on the development of 3D-printed shape-programmable magneto-active soft materials (MASMs) for biomimetic applications^143^ (Figure 3D). They utilized an FFF 3D printer to fabricate soft actuators that can undergo fast, reversible, and programmable shape transformations under uniform magnetic fields (UMF). The MASMs were composed of a flexible silicone rubber matrix embedded with oriented magnetic structural elements, allowing for precise control over the magnetization profiles. The study demonstrated the creation of various biomimetic structures, such as an inchworm, manta ray, and soft gripper, which could perform complex motions, including walking, swimming, and gripping. The 3D printer used in the study was custom-designed for the fabrication process, enabling the precise orientation of magnetic elements necessary for the desired shape transformations.

Li et al. reported on the development of multilayer magnetic miniature soft robots with programmable magnetization for biomedical and other advanced applications.^141^ They utilized a DLP 3D printer to fabricate various multilayer 3D structures, including stool-shaped, gripper-shaped, capsulelike, helical, and walking robots. The robots were composed of UV-curable polymer matrices embedded with magnetically hard or soft nanoparticles, which were precisely oriented by using a magnet during the printing process to achieve discrete magnetization profiles. This enabled the robots to perform complex movements such as gripping, rolling, swimming, and walking under an applied magnetic field. The DLP-based 3D printer was custom-designed to accommodate the magnetic encoding and multilayer fabrication process, which significantly enhanced the structural complexity and functional capabilities of the printed robots compared to previous methods that primarily produced 2D structures.

Cao et al. reported on the development of 3D-printed magnetic actuators designed for biomimetic applications^144^ (Figure 3E). They employed a FFF 3D printer to fabricate various actuators by using a new printable magnetic filament composed of thermoplastic rubber (TPR) material and magnetic particles. These actuators demonstrated programmable shape transformations in response to magnetic fields, imitating the motion characteristics of natural creatures, such as octopus tentacles, butterflies, and flowers. The study highlighted the use of finite element method (FEM) simulations to predict and guide the deformation behavior of the actuators, showcasing the potential of magneto-active materials in soft robotics, biomedicine, and bionics. The FFF 3D printer utilized was a commercial model, customized for this fabrication process to ensure the precise creation of complex, responsive structures.

Ansari et al. reported on the development of small-scale soft robots with programmable magnetization for applications requiring multimodal locomotion.^148^ They employed a custom-extrusion-based 3D printing method that integrates magnetic anisotropy directly into the printed soft structures. The magnetic ink, composed of a UV-curable resin and neodymium iron boron (NdFeB) particles, was extruded while being subjected to a magnetic field generated by a custom electromagnetic coil system. This setup allowed for the precise orientation of the magnetic particles in the ink, enabling the creation of soft robots with complex and on-demand magnetization profiles. The resulting structures demonstrated the ability to perform various actuation tasks, such as twisting, traveling in narrow spaces, and folding into 3D shapes.

Cao et al. reported on the development of ultraflexible magnetic actuators for soft robotics applications, utilizing a novel 3D printing strategy based on screw extrusion technology.^147^ The actuators were fabricated by using a material composed of carbonyl iron particles embedded in a TPR matrix, enabling programmable deformation under magnetic fields. The 3D printer employed was custom-built to address the challenges of printing low-modulus materials and ensuring continuous feeding and precise extrusion. The actuators demonstrated various biomimetic functions, such as adhering, releasing, and pumping, with their performance validated through both experimental tests and finite element analysis.

#### 3.2.3 Thermally Actuated Robotics

Thermally responsive materials change their shape, size, or properties when exposed to temperature changes. One-way thermally responsive polymers can change shape upon heating or cooling but require external intervention, such as manual resetting, to return to their original form after each actuation. In contrast, two-way thermally responsive polymers represent significant advancement. They can repeatedly and automatically cycle between two shapes in response to temperature fluctuations without any external resetting, allowing for continuous, seamless motion, making them suitable for soft robotics.^149–151^ This feat is primarily achieved through melting-induced contraction and crystallization-induced elongation, key processes facilitated by the phase transitions between crystalline and amorphous states within the material. Such transitions are underpinned by entropy elasticity, wherein alterations in temperature prompt shifts between these phases, driving the material to contract and elongate cyclically. By systematically cycling the temperature between the melting and crystallization points, the material can autonomously undergo repetitive actuation, demonstrating remarkable self-sufficiency in its functionality. For example, a shape-shifting electrode,^152^ electrothermal activated,^153^ shape transformation induced grasping and locomotion,^154^ flora mimic,^155,156^ enabling 4D textiles,^157^ printing of bioinspired lattice metamaterials,^158^ controlled origami,^159^ and thermally activated self-healing,^160,161^ as presented in the following examples and in Figure 3.

Zarek et al. reported on the development of 3D-printed SMPs for flexible electronic devices^152^ (Figure 3F). They utilized an MSLA 3D printer to fabricate complex SMP structures from a methacrylated semicrystalline polymer, enabling high-resolution printing and precise shape memory behavior. The material exhibited excellent shape memory performance, with a strain recovery rate of >93% and a strain fixity rate greater than 98%. The SMPs were integrated with conductive materials to create responsive electrical devices such as temperature sensors and shape memory connectors. In another work, Wang et al. reported on the development of electrothermally controlled origami structures fabricated using 4D printing of continuous fiber-reinforced composites^153^ (Figure 3G). They employed an FFF 3D printer to create origami with integrated carbon fibers, which enabled precise shape-shifting through localized Joule heating. The composite material, composed of continuous carbon fibers (CCFs) and SMP, exhibited significantly enhanced mechanical properties and a uniform thermal distribution, allowing for precise control of the shape recovery process. The study demonstrated various applications of the electrothermal origami, including reconfigurable robots, customizable materials, and programmable wings, showcasing the potential for multiscenario and multitask applications. Cortes et al. fabricated 4D-printed nanocomposites by DLP using mixtures of acrylated/methacrylated resins doped with 0.1 wt% carbon nanotubes. After UV and thermal postcuring, the printed materials exhibited tunable mechanical properties and glass transition temperatures (*T*_g_ from 15 to 190 °C). Shape memory behavior was activated by heating above *T*_g_ using a conventional oven or by localized infrared (IR) irradiation, where CNTs enhanced the heat absorption and accelerated the recovery. Simple actuator-like structures were fabricated, which could reversibly bend and recover shape under thermal or IR stimulation. Memory activation was achieved through both conventional oven heating and infrared (IR) radiation, with IR providing significantly faster recovery times due to enhanced absorbance from the nanotubes.^162^

Yang et al. reported on the development of 3D-printed photoresponsive devices based on shape memory composites for advanced materials applications.^156^ They utilized an FFF 3D printer to fabricate devices from a composite material composed of PU mixed with carbon black (CB), which provided high photothermal conversion efficiency. The devices exhibited shape memory behavior triggered by external light sources, including natural sunlight. The study quantified the effects of material thickness and light intensity on the shape recovery process, demonstrating that 3D-printed devices could be used in applications such as biomimetic sensors and soft robotics. Wang et al. reported on the development of biomimetic shape–color double-responsive 4D printing for advanced material applications.^155^ They utilized an FFF 3D printer to fabricate composites made of PLA and thermochromic pigments (T-PIGs), enabling simultaneous shape transformation and color change in response to temperature stimuli. The study demonstrated the ability to control both the speed of shape recovery and color transition by adjusting printing parameters, such as nozzle temperature, nozzle height, and geometric thickness. The printed structures, including a blooming flower and camouflaging octopus, showcased complex, programmable responses and highlighted the potential for applications in soft robotics and intelligent materials.

Kuang et al. reported on the development of highly stretchable, shape-memory, and self-healing elastomers designed for 4D printing applications.^161^ They utilized a DIW 3D printing technique combined with UV-assisted curing to fabricate a semi-interpenetrating polymer network (semi-IPN) elastomer. The material was composed of a urethane diacrylate (AUD) matrix embedded with semicrystalline polycaprolactone (PCL), which served dual roles as the shape-memory switching phase and the self-healing component. Invernizzi et al. reported on the development of a 4D-printed thermally activated self-healing and shape memory polymer (SMP) based on PCL for soft robotics and other advanced applications.^160^ They used a DLP 3D printer to fabricate objects from a novel material combining polycaprolactone dimethacrylate (PCLDMA) with 2-ureido-4­[1*H*]-pyrimidinone (UPy) motifs. This combination provided the material with both shape memory and self-healing properties, with the self-healing triggered by thermal activation. The study demonstrated the printability of the material by producing a shape-changing actuator, such as an opposing thumb, that could recover its original shape even after being damaged and healed. The mechanical properties of the printed objects were found to be comparable to those of conventional PCL-based materials, making them suitable for applications in soft robotics and human–machine interaction.

#### 3.2.4 Photo-Actuated Robotics

In photoresponsive systems, various mechanisms, including photothermal, isomerization, and greyscale, dictate the mode of motion or actuation.^163^ A crucial component in these systems is a light-sensitive element that reacts to incident light, yielding either mechanical or thermal energy. In photothermal responsive materials, the composite typically comprises a proficient thermal conductor coupled to an effective light absorber. First, in photothermal mechanism, black materials, such as carbon derivatives, excel at absorbing light and subsequently elevating their temperature.^164^ Similarly, metal nanoparticles resonate with the incident light, inducing plasmonic heating, while dark polymers such as polypyrrole demonstrate remarkable performance in photothermogenesis too.^165,166^ These programmed localized heating leads to mechanical deformations at designed areas, raising directional actuation, which represents their potential for soft robotics, as shown in the representative examples in Figure 3. In addition, Some examples include fin-ray locomotion,^167^ sophisticated programmable optomechanical actuation enabled by combination of biopolymer material (silk fibroin) and a reconfigurable photonic crystal structure,^165^ polyurethane-urea based elastomer bidirectional actuation driven by photochemical and photothermal coupling mechanism,^168–171^ reprogrammable LCE photoactuators,^172^ fast near-infrared light responsive actuation,^173^ biomimetic locomotions,^174^ and plasmonic enhanced photothermal responsive.^175^ For example, Wu et al. reported the development of a tunable light-responsive polyurethane–urea elastomer driven by a photochemical and photothermal coupling mechanism for soft robotics and biomedical applications^168^ (Figure 3H). The elastomer, named PAzo, was synthesized by incorporating azobenzene derivatives into the main chain of poly­(ε-caprolactone)-based polyurethane urea (PCL–PUU), resulting in a material that exhibits controllable stiffness and softening under visible light. The PAzo elastomer demonstrated exceptional hyperelasticity with a stretchability of 575.2% and a strength of 44.0 MPa. A bilayer actuator composed of PAzo and polyimide films was developed to showcase tunable bending modes by varying the intensity of incident light. Montesino et al. reported on the development of reprogrammable 4D-printed LCE photoactuators utilizing light-reversible perylene diimide radicals.^172^ These LCE actuators, fabricated using a DIW 3D printer, can morph into different 3D shapes under light stimuli, driven by a combination of photothermal and photochemical effects. The actuators were printed with an ink containing a perylene diimide chromophore, which allows the material to change shape in response to green light and then reconfigure under far-red light. This dual-light-responsive behavior enables complex, reconfigurable actuation without the need for structural modification of the actuator. Xia et al. reported the development of a 4D-printed bionic soft robot inspired by starfish, designed for applications requiring rapid responses to NIR light^173^ (Figure 3I). The robot, composed of reduced graphene oxide-poly­(*N*-isopropylacrylamide) hydrogel (rGO-PNH), was fabricated using DIW 3D printer, allowing precise control over the hydrogel’s mechanical properties and photothermal conversion efficiency. The actuator demonstrated fast bending and orientation toward the light source within 20 s of exposure to NIR light, mimicking the predatory behavior of a starfish.

Lou et al. reported the development of a photothermal-driven crawlable soft robot with a bionic earthworm-like bristle structure.^174^ The robot, composed of three main parts, two bionic bristle units, and a central LCP actuator, was designed to mimic the crawling motion of an earthworm. The LCP actuator responds to NIR light by contracting and expanding, while the bionic bristles provide directional friction, allowing the robot to crawl forward on a flat surface. The study demonstrated that the soft robot could achieve a maximum crawling speed of 4.4 mm/min under the optimal conditions. This novel design, which does not require complex 3D deformation or specific environmental conditions, highlights the potential of photothermal-driven actuators in the development of remote-controlled soft robots for various applications.

Skillin et al. reported the development of thick 3D-printed LCE nanocomposites designed for photothermal actuation in soft robotics.^175^ The LCE nanocomposites were fabricated by using a DIW 3D printer, which allowed for precise alignment of the LCE molecules along the print path. The nanocomposites were enhanced with gold nanorods (AuNRs) that were dispersed by using a two-step ligand exchange process with poly­(ethylene glycol) (PEG) thiol, significantly improving their photothermal efficiency. The study demonstrated that these 3D-printed LCE-AuNR actuators could achieve rapid actuation speeds of over 60% strain per second when exposed to NIR light. The actuators were also capable of performing complex deformations such as contraction and twisting, highlighting their potential for applications in soft robotics and medical devices.

In photon-isomerization, molecules capable of cis–trans transitions in response to light play a pivotal role.^176,177^ This process hinges on the fundamental principle of transitioning between cis and trans configurations upon illumination, leading to alterations in packing density within the material.^178,179^ Consequently, this shift in packing density induces changes in the overall volume of the material, generating and/or removing the mechanical energy in the form of internal strain. Sartori et al. reported the development of a 4D-printed LCE swimmer^178^ designed for biomimetic applications, specifically mimicking the propulsion mechanisms of underwater organisms like ephyra, an early developmental stage of jellyfish. The swimmer, composed of four lappet-like structures, was fabricated using extrusion printing with azobenzene-containing photopolymerizable inks. These LCEs exhibit rapid and significant photomechanical responses to moderate-intensity UV and green light, enabling the swimmer to propel itself underwater by synchronous lappet bending. Keutgen et al. reported the development of mesoscopic microgels^179^ with precisely positioned supramolecular recognition motifs, designed as soft building blocks for assembly and light-triggered disassembly in soft robotics applications. The microgels, fabricated using 3D stereolithographic printing, were composed of a photoresist material including 2-hydroxyethyl acrylate, ethylene glycol diacrylate, and phenyl bis­(2,4,6-trimethylbenzoyl) phosphine oxide, with azobenzene (Azo) and α-cyclodextrin (αCyD) used as the supramolecular recognition motifs. The microgels could reversibly assemble into larger structures or disassemble under light stimuli, demonstrating the potential for reconfigurable structures in soft robotics.

#### 3.2.5 Comparison of Active Materials for Soft Robotics

The selection of active (smart) materials for soft robotics is dictated not only by their responsiveness to external stimuli but also by their processability, actuation performance, and compatibility with 3D printing strategies. Each class of stimulus-responsive materials discussed above offers unique benefits and limitations, depending on the application requirements and printing constraints.

Electroactive materials are widely used in soft robotics for their fast, electrically driven actuation behavior and high spatial precision. This class includes DEAs, ionic polymer–metal composites, and electroactive hydrogels, which have been fabricated using DIW, MSLA, and multimaterial FFF. These materials provide rapid, reversible actuation and allow fine control of deformation via electric field strength and electrode design, enabling soft grippers, bioinspired locomotion systems, and haptic interfaces. Their main advantages are high actuation resolution, fast response times, and potential for embedded sensing-actuation integration. However, drawbacks include high voltage requirements (especially for DEAs), limited durability due to material fatigue under cyclic loading, environmental sensitivity (notably for ionic polymer–metal composites), and complex multilayer or electrode fabrication processes, and they are often limited to lightweight devices only. They are typically best suited for low-load applications where fast, controllable motion is prioritized.

Magnetoresponsive materials enable remote, untethered actuation through the use of external magnetic fields, offering an attractive solution for soft robots operating in confined or submerged environments. These systems are typically composed of thermoplastic rubber, UV-curable acrylates, or silicones embedded with magnetic fillers, such as neodymium iron boron (NdFeB), iron oxide (Fe_3_O_4_), or carbonyl iron particles. Printing strategies include FFF, DLP, and extrusion-based methods combined with magnetic field alignment during fabrication. Key benefits include wireless control, programmable shape transformations via magnetic domain encoding, and compatibility with diverse geometries and printing strategies. However, implementation challenges include the need for custom-built printing setups to align magnetic domains, relatively low force output compared to electrical or thermal actuators, material fatigue under repeated cycling, and limited effectiveness over large distances. Despite these, magnetoactive systems remain unmatched in applications requiring versatile untethered movement and environmental adaptability.

Thermally responsive materials are a staple of 4D printing due to their ease of activation and broad range of mechanical tunability. Common examples include SMPs, PCL and PUAs derivatives, often enhanced with photothermal fillers like CNTs or carbon black. These materials are compatible with DLP, FFF, DIW, and MSLA 3D printing platforms. Advantages include high strain recovery, reprogrammable shape transformations, and the ability to integrate self-healing capabilities or dual-stage actuation through melting-induced contraction and crystallization-induced elongation. However, thermal actuation is relatively slow and may require external heating sources, and most systems offer only one-way actuation, unless specifically designed for two-way cycling. These materials are ideal when controlled, time-based motion or long-term structural reconfiguration is prioritized over actuation speed or remote operation.

Photoresponsive materials provide light-triggered actuation mechanisms for soft robotics, enabling contactless and spatially selective motion. These systems rely on photothermal or photoisomerization mechanisms and are typically composed of reduced graphene oxide-poly­(*N*-isopropylacrylamide) hydrogels, gold nanorod-enhanced LCEs, or azobenzene-functionalized polyurethane–urea elastomers. Fabrication is commonly performed using DIW, SLA, or stereolithography. Depending on their composition, they respond to UV, visible, or near-infrared light. Their key strengths include remote activation, programmability, and the ability to induce localized or reconfigurable deformation, ideal for applications like locomotion or complex shape change. Limitations include relatively low actuation forces, slower response compared to electrical systems, dependency on filler dispersion and geometry for photothermal efficiency, and the need for a precise optical setup. These materials are especially useful in systems that require wireless, spatially resolved control without the need for embedded electronics.

Based on the discussion in this section and the examples presented, Table 2 compares the main smart material classes used in soft robotics, including their representative materials, printing methods, and key advantages and limitations. While each class has its own strengths and drawbacks, an understanding of these distinctions is essential when selecting an actuation mechanism during the design of a soft robotic system.

## 4 Design Approaches for Soft Robotics

The role of design in the fabrication of soft robots through 3D printing is pivotal, as it dictates the functionality, adaptability, and efficiency of the resulting robots. Design of a soft robot is a process of determining its geometry, material, and functional features to meet the predefined functionality and reliable performance under specific operating conditions. At its core, the design process is about selecting the optimal combination of form, materials, and function. Various design approaches have been developed to leverage the unique capabilities of 3D printing technologies. As soft robotics is a rapidly evolving field, a standardized definition of design approaches is still lacking. Therefore, in this report, we attempt to classify the diverse approaches found in the scientific literature into five broad categories, as presented in Figure 4: (a) bioinspired, designs inspired by structures or mechanisms found in nature; (b) biohybrid, designs that incorporate biological components alongside engineered elements; (c) metamaterial-based, designs that utilize architected materials with unusual deformation or transformation properties to enable soft robotic actuation; (d) multimaterial, design which integrates different materials in a single print to achieve complex mechanical properties and functionalities, (e) functional, designs that begin with a specific functional goal and leverage materials, geometries, and fabrication methods to fulfill that objective. It is worth noting that these categories are not mutually exclusive and several examples could reasonably belong to multiple groups. For clarity, in this review, each example has been classified based on its primary design strategy, as emphasized in the original publication. While the focus remains on additive manufacturing, we included a few non-AM examples where the design concept is particularly illustrative and highly relevant.

**Figure 4:**
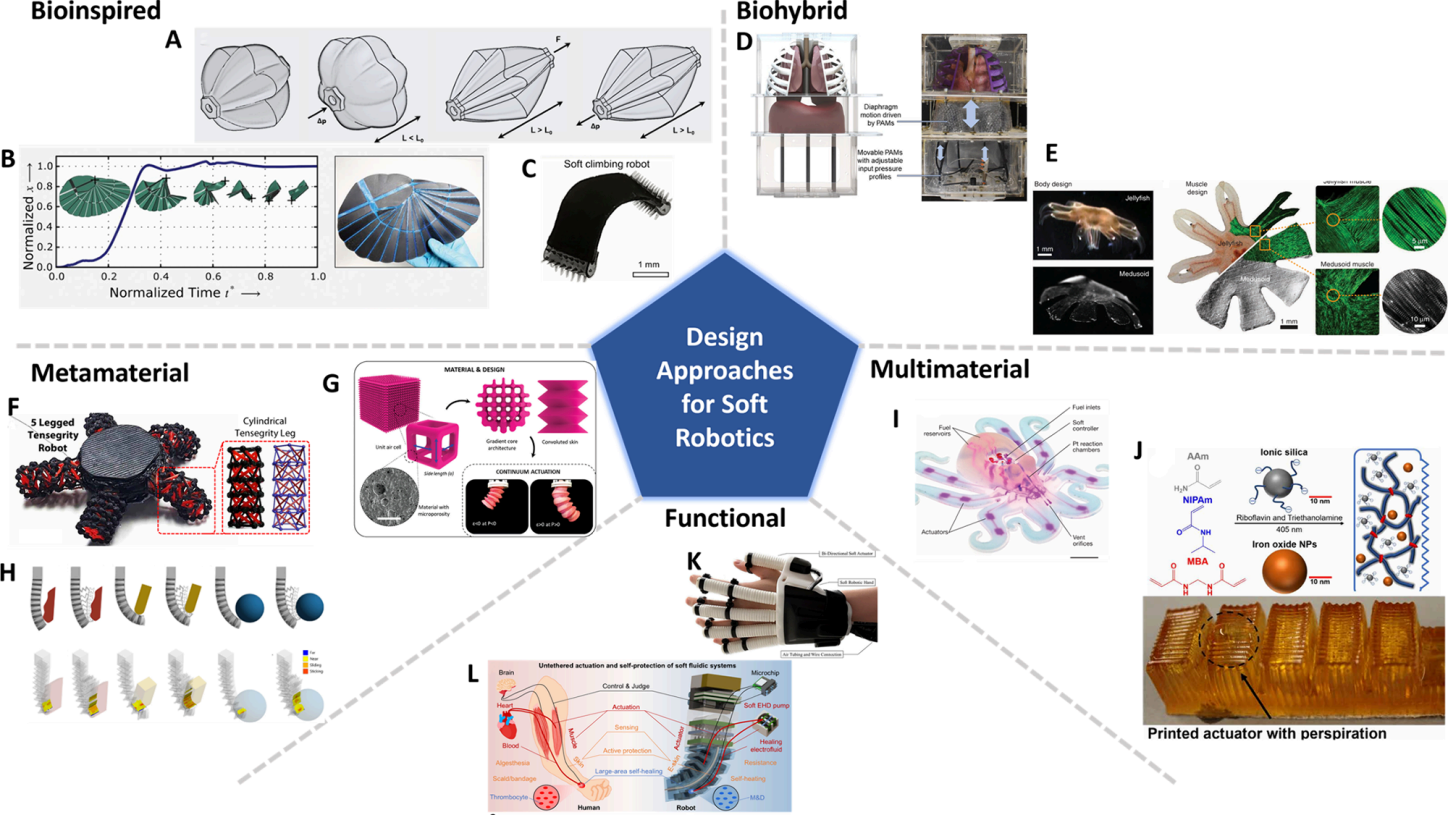
Design approaches principles for soft robotics. Bioinspired. (A) Geometry-based pleated GRACE pneumatic muscles that bidirectionally contract/elongate. Reproduced with permission from ref 79. Copyright 2022 American Association for the Advancement of Science. (B) A self-folding origami wing inspired by earwigs, adding spring elements for full, robust folding. Reproduced with permission from ref 182. Copyright 2018 American Association for the Advancement of Science. (C) Wireless ferromagnetic millirobot with structured footpads that climbs diverse 3D surfaces. Reproduced with permission from ref 183. Copyright 2022 under Creative Commons Attribution License 4.0 (CC BY) American Association for the Advancement of Science. Biohybrid. (D) Biohybrid respiratory simulator, 3-D printed rib cage plus pneumatic drives and explanted lungs. Reproduced with permission from ref 192. Copyright 2020 AIP published under Creative Commons Attribution License 4.0 (CC BY). (E) Tissue-engineered jellyfish robot with silicone body and rat-muscle bilayer for medusoid propulsion. Reproduced with permission from ref 193. Copyright 2012 Springer Nature. Metamaterials. (F) 3D printed five-leg tensegrity robot, monolithic tendons and magnets drive the bending/expansion gait. Reproduced with permission from ref 197. Copyright 2020 American Association for the Advancement of Science. (G) Elastic pneumatic actuator achieving two-way bending from one pressure line. Reproduced with permission from ref 204. Copyright 2023 Wiley-VCH, under Creative Commons Attribution 4.0 International License 4.0 (CC BY). (H) TPU 3D printed metamaterial lattice enables conformal surface gripping. Reproduced with permission from ref 237. Copyright 2021 The Author(s). Published by Frontiers Media SA under the Creative Commons Attribution 4.0 International License (CC BY 4.0). Multimaterials. (I) Fully soft autonomous robot powered by catalytic combustion and onboard soft circuits. Reproduced with permission from ref 95. Copyright 2016 Springer Nature. (J) Hydrogel-based soft robot that sweats via pore opening above 30 °C for self-cooling. Reproduced with permission from ref 214. Copyright 2020 American Association for the Advancement of Science. Functional. (K) Directional-actuated soft robotic hand for patient hand rehabilitation. Reproduced with permission from ref 221. Copyright 2023 Frontiers, under Creative Commons Attribution 4.0 International License 4.0 (CC BY). (L) Self-protecting fluidic soft robot: damage-sensing chip triggers healing electrofluid flow. Reproduced with permission from ref 224. Copyright 2023 Springer Nature, under Creative Commons Attribution 4.0 International License 4.0 (CC BY).

To better frame the design strategies discussed in this section, it is important to emphasize their close relationship with the materials and fabrication technologies introduced in sections 3 and 4. The feasibility and performance of each design approach are often constrained or enabled by the available materials and compatible additive manufacturing methods. For example, architected metamaterial designs typically require high-resolution processes such as DLP or SLA, and depend on materials with low hysteresis and high resilience to preserve programmed mechanical responses. Biohybrid systems, on the other hand, demand biocompatible and cell-supportive substrates, such as hydrogels or elastomers, that can interface with living tissues. Multimaterial strategies require good intermaterial adhesion and compatibility, while bioinspired and functional designs vary in their material demands depending on the targeted performance, such as stretchability, compliance, or thermal stability. Throughout this section, examples are selected to illustrate design logic and reflect how material selection and fabrication methods shape the final robotic system.

### 4.1 Bioinspired Designs

Bioinspired design involves creating systems and devices that mimic the structures, functions, and mechanisms found in nature. In soft robotics, this approach draws inspiration from biological organisms to develop flexible, adaptive, and efficient robotic systems, as shown in Figure 4. For example, Whitesides group demonstrated fabrication of octopus inspired robotic tentacles,^180^ fabricated using silicones of different stiffness by casting inside 3D printed molds. The tentacles could achieve a wide range of motion and manipulate objects with complex shapes. The tentacles could also be equipped with cameras, fluid delivery system, and a suction cup for added functionality. Finite element modeling was used to estimate with fair accuracy, the expansions of micropneumatic channel for increasing pressures. Mazzolai’s group designed biomimetic artificial muscles^79^ using soft actuators made of TPU elastomers (Figure 4A) that could contract and elongate, enabling diverse actuation modes. The unique design of the artificial muscles enables contraction and elongation upon pressure variation. The design can be mathematically modeled, and the actuators can be fabricated at different scales and materials by 3D printing. The artificial muscles have excellent load bearing capacity, where the 4 cm long actuators could lift weights of 8 kg at 3 bar pressures. Paik’s group demonstrated vacuum powered soft pneumatic actuator system (V-SPA)^181^ powered by a single shared vacuum power supply, enabling multidegrees of freedom actuation. The vacuum actuators were manufactured by coating open-celled PU porous foam materials with silicone rubber. When multiple V-SPA are connected in series and operated, they could achieve locomotion on flat surfaces, walls, and also pick and place objects like robotic manipulators. With the addition of jamming pillars (comprising of grounded coffee powder) to V-SPA, the stiffness of the soft robot also was modulated.

Studart’s group utilized FFF 3D printing technique to 3D print bioinspired spring origami structure^182^ (Figure 4B) that can function like the wing of an earwig. The design takes inspiration from earwig wings, which display incompatible folding patterns, remain open during the flight, and self-fold rapidly without muscle actuation. The robots include acrylonitrile butadiene styrene (ABS) for the stiff polymer facets and thermoplastic polyurethane (TPU) for the rubber-like hinges. The 3D printing assisted in fabricating the wing in the folded state, allowing a greater degree of customization of the interfacet angle. As a result, by exploiting both geometrical and material properties, the origami structures were able to achieve fast morphing (within 80 ms) triggered by environmental stimuli. Soft robotic devices for surgery and drug delivery require controlled locomotion on soft and wet surfaces. To address this challenge of soft robot locomotion, Wu et al. designed a polydimethylsiloxane (PDMS) based soft robot (Figure 4C) actuated by a magnetic field, that relies on peeling-and-loading mechanism,^183^ which allows both soft body deformation (shape morphing behavior) and whole body motion (rigid-body translation) of robot under external magnetic fields. They further integrated microstructured adhesives and tough bioadhesives on the footpad of the robots to achieve controllable adhesion and force to climb soft and wet surfaces, including porcine tissues. Appropriate variation in the magnetic field triggered, pinning, peeling, and translation of the soft robot for controlled locomotion in complex terrains.

In another report, Tsai et al. designed miniature soft jumping robots^184^ fabricated using projection additive manufacturing. The four-bar linkage robot is made of a carbon EPU41 elastomer and is assembled with a rigid polymer latch, a rubber band, and a coiled artificial muscle. Inspired by the kinematics of the locust jumping mechanism, the robot stores elastic energy throughout its body and releases it as kinetic energy, enabling it to jump up to 60 times its body length. The jumping mechanism was driven by the coiled artificial muscles connected to a latch trigger. Jiang et al. also designed a pipe climbing robot^185^ made of origami clutches and soft modular legs for automation in hazardous environments. The robot could climb both within the pipes and on the pipes and up to 45° bending angles and on variety of materials including PVC, rubber, and metals. Robot consisted of soft linear actuator for movement, two origami clutches for multi degrees of freedom motion, and two pairs of soft modular legs for climbing. The pneumatic actuators were made of soft silicones, while structural origami components were 3D printed using thermoplastic urethane.

Gu et al. demonstrated a soft wall climbing^186^ robot made of dielectric elastomer artificial muscles. The artificial muscles consisted of prestretched dielectric elastomer membrane (VHB 4910) sandwiched between compliant carbon grease electrodes and attached to electroadhesive feet consisting of copper electrodes sandwiched between polyimide films. Wall climbing was achieved by synergistic control between deformation of the robot body (controlled by applied voltage to dielectric elastomer) and controlled adhesion of the robot feet. The robot is capable of climbing variety of walls made of glass, paper, and wood at 90° and moving at 0.75 body lengths per second, including climbing, crawling, and turning motions. A similar dielectric elastomer actuated soft robot with reconfigurable chiral lattice foot^187^ was designed by Gu’s group. The robot consisted of a chiral lattice foot and a flat foot which enables immediate and reversible forward, backward, and circular directional changes during directional movement. These multimodal movements are achieved from dynamic resonant and chiral twisting effects, which are intrinsically embedded in the lattice structural design. The left, right, or circular movement of the robot was triggered by changing the frequency of the voltage, resulting in relative motion of the lattice foot and the flat foot, and hence the multidirectional movement. Sun et al. fabricated a fully 3D printed tortoise^188^ like amphibious soft mobile robot that can move on hard and soft surfaces and also in water. The robot used a bionic tortoise leg actuator made of photocurable stretchable polyurethane elastomer (Shore 40 A) and fabricated by digital light processing. The design of the actuator enabled simultaneous bending of the actuator in both directions, which simplified the robot control for movement.

In another report, a soft swimming robot^189^ mimicking the breast stroke swimming maneuver of human beings was demonstrated by using dielectric elastomers for actuation. A special structure of the swimming leg of the robot allows for self-adaptation during swimming to increase forward propulsion. During dielectric elastomer actuation, the two legs of the robot form a semiclosed space and self-adaptive feet change its water facing direction depending on the water pressure. As a result, the robot with self-adaptive feet could achieve 3.15 times faster swimming speeds at 0.77 body lengths per second. The super light robot weighing only 14.3 g was made of 188 μm polyethylene terephthalate frame, 38 μm flexible adaptive foot, and two layers of dielectric membranes (VHB 4910, 3M) connected to carbon grease electrodes. Wang et al. designed a non-Eucledian-plate^190^ underwater soft robot inspired by jellyfish using liquid crystal elastomers. The soft robot consists of a 3D printed non-Euclidian plate, designed with archimedian orientation, that deforms in contact with organic solvents. The robot consists of an LCE embedded with MWCNTs which have high photothermal conversion efficiency for NIR radiation. The autonomous deformation is caused by release of internal stress. When coupled with NIR illumination, the organic solvent inside the robot vaporizes, releasing bubbles and hence generating propulsion. The unique robot shows diverse locomotion modes including climbing walls, jumping, turning, rolling, and flipping in a variety of organic solvents and opens up new design paradigms using 4D printing strategies.

Hiramandala et al. designed a hedgehoginspired soft robot companion that uses acupuncture and acupressure principles to facilitate relaxation for the user.^191^ The robot was made of assembly of 3D printed silicone elastomers including the quills of the robot that provide acupuncture and acupressure sensation to the user.

### 4.2 Biohybrid Designs

Biohybrid design combines biological tissues or cells with synthetic materials to create systems that combine the benefits of both living organisms and robotics. In soft robotics, as presented in the examples at Figure 4, biohybrid designs harness the functionality of biological components to achieve advanced movement or responsiveness. Horvath et al. designed an organosynthetic (Figure 4D) soft robotic respiratory simulator^192^ to function as a reliable testbed for devices and reduce the need for animal testing. The simulator was constructed by fabricating a high-fidelity anthropomorphic model of the diaphragm using thermoplastic elastomeric materials and integrating pneumatic artificial muscles programmed to move in a clinically relevant manner. Organic lungs were inserted into the thoracic cavity of the model to verify that the inflation and deflation caused by the artificial muscles induced the desired exhalation and inhalation. This was confirmed using integrated sensors that measured the pressure, volume, and flow rate of the gases.

In two important reports by Parker’s group, soft bioinspired soft robots Parker’s group designed by reverse engineering (Figure 4E) a jellyfish robot^193^ from chemically dissociated rat tissue and silicone polymer. The freely swimming jellyfish robot was designed based on computer simulations and experiments to match the jellyfish swimming abilities, by quantitatively mimicking structural design, stroke kinematics, and animal fluid interactions. The reverse engineering was accomplished by using a sheet of cultured muscle tissue triggered by an electric field that achieves a complete bell contraction as that in jellyfish. Further, the power and recovery strokes of jellyfish were mimicked using a bilayer of muscle and synthetic elastomer. In another report, the same group fabricated a biohybrid^194^ ray fish that can swim and phototactically follow a light cue. The fish was fabricated by patterning dissociated rat cardiomyocytes on an elastomeric body enclosing a microfabricated gold skeleton. The tissue-engineered ray is capable of muscle contraction in the downward direction, while the rebound movement is achieved from the elastic energy stored in the gold skeleton. The myocytes on the elastomeric body are engineered to be responsive to light. Thus, by controlling the modulating light frequency, the speed of the robot fish was controlled, while the direction of the motion was controlled through independently eliciting right and left fins. Although not fabricated via additive manufacturing, these two soft robots developed serve as inspiring demonstrations of the biohybrid design principles, integrating living muscle tissues with synthetic scaffolds to achieve functional soft robots.

### 4.3 Metamaterial Based Designs

Metamaterials are engineered materials whose properties are determined more by their structure than their material’s composition. Unlike traditional materials, their behavior can be precisely controlled by designing their architecture,^195^.^196^ Lee et al. demonstrated the benefits of using tensegrity structures in soft robotics. These structures were fabricated using a combination of 3D printing and sacrificial molding.^197^ A sacrificial mold with internal channels was printed using PVA, while the struts were printed with PLA. Polymeric smart materials were then injected into the mold and thermally cured, and the mold was dissolved in water. By selection of appropriate soft and stiff elements and adjustment of design parameters such as geometry, topology, density, coordination number, and complexity, the system-level mechanics of the soft structures could be programmed. Utilizing these advanced techniques, the authors designed a five-legged robot (Figure 4F) capable of walking in any direction and tensegrity actuators created through an algorithmic design approach. Mark et al. developed a design methodology for multidegree of freedom soft actuators using geometric origami^198^ patterns. The method employed a generalized design approach to create various cylindrical origami patterns, including Kresling, cylindrical Miura, Yoshimura, and Accordion. They demonstrate, using a TPU 95A filament, that a 3-DOF pneumatic actuator capable of bending and expansion can be constructed by a simple superimposition of two cylindrical origami patterns resulting in separation of chambers inside the module. By carefully selecting dimensions, they optimized force generation at lower strains. Moreover, these designs were 3D printed without requiring support structures, allowing for a streamlined single-step fabrication.

Kirigami is an interesting approach to designing soft robots where morphing characteristics of the programmed geometries can be exploited to achieve the desired spatial configuration. Gladman et al. utilized kirigami designs along with programmed anisotropy to 4D print hydrogel-based shape morphing soft robots.^199^ The photocurable hydrogel consisted of an aqueous solution of *N*,*N*-dimethylacrylamide, photoinitiator, nanoclay, glucose oxidase, glucose, and nanofibrillated cellulose (NFC). The hydrogel readily swells in water, leading to shape morphing soft robots. By properly programming the alignment of cellulose fibrils, the desired morphing behavior could be generated, showcasing the use of smart design and materials to fabricate soft robots in various flower geometries. Moreover, Jin et al.,^200^ Hong et al.,^201^ and Kang et al.^202^ demonstrated excellent examples of kirigami-based metamaterial designs for programmable shape morphing and high-strength gripping. These works highlight innovative structural strategies for soft robotics. However, none were fabricated by using additive manufacturing.

In soft grippers, handling heavy soft and thin objects is challenging due to trade-off between compliance, strength, and precision. Song et al. developed a soft adhesive interface for soft robots that enables high load carrying capacity through gecko inspired surface adhesion.^203^ Their invention attempted to address the trade-off between the 3D surface conformability and the adhesion strength of typical gecko-inspired membranes. The system comprised a gecko-inspired elastomeric made by a siloxane based curing agent (Sylgard 184) microfibrillar adhesive membrane supported by a pressure controlled deformable gripper body. The change in internal pressure aids the adhesion, and as a result of which the adhesion force increased 14 times compared to the conventional systems.

In another report, Joe et al.^204^ introduced a novel class of soft robotic actuators by leveraging a metamaterial design based on combined microporosity and macroporosity, achieved through a single-step DLP 3D printing of stretchable polyurethane emulsions (Figure 4G). The printed elastic lattices exhibit tunable stiffness and high deformability, enabling jointless continuum structures that encode biaxial, axial, and bidirectional bending motions. This approach demonstrates how material-level porosity and structural-level tessellation can be co-engineered to produce soft robots with programmable multidimensional kinematics in a monolithic architecture. Another interesting work was made by Tawk et al., who developed a fully 3D printed soft modular gripper using FFF with TPU, integrating mechanical metamaterial auxetic structures with compliant ribs directly into the soft fingers^71^ (Figure 4H). This combination enhanced the conformability and stability of the gripper by increasing the contact area and reducing the pressure during grasping. Their design showcases how material selection and structural patterning at the metamaterial level can significantly improve the grasping performance in soft robotics. Shepherd’s group designed a human heart-shaped fluidic pump that can pump water at flow rates of more than 430 mL/min at a low pressure difference of around 14 kPa.^205^ The pump consisted of poroelastic foams made by heating a silicone and ammonium hydrogen carbonate mixture. Nylon mesh was embedded within the foam before sealing to program the actuator motion. Further, the pump provided sustained flow rates in contrast to sudden bursts of flow furnished by the several reported combustion powered pumps.

### 4.4 Multimaterial Design

Often to achieve global compliance while maintaining conformal deformation of a soft robotic, the robot is designed with both rigid and soft materials.^206^ Particularly in the case of soft robotic grippers, incorporating sensors can augment its functionality, and multimaterial 3D printing can significantly support such designs. In one such application, a multimaterial based origami gripper^207^ with a tactile sensor was fabricated by FFF printing, using a digital material (DM) filament. The DM filament was fabricated by combining multiple base material filaments. When the DM filament is extruded through an FFF printer, spatial programming of properties such as mechanical strength, electrical conductivity, and color is achieved. Alici’s group demonstrated 3D printed omnipurpose soft gripper that can grip objects with varying stiffness, weight, size, and shape.^71^ The soft gripper was printed by using a commercial FFF 3D printer. Each finger could achieve a blocking force of more than 30 N and worked for more than 26000 cycles. The blocking force generated by the grippers was estimated by both finite element modeling and analytical model (within 1.7% error) approaches. The gripper utilizes a bundle of linear soft vacuum actuators that produce a linear stroke motion to pull tendon-driven soft fingers. Additionally, the gripper is equipped with a suction cup to assist in grasping heavier objects. Thermoplastic polyurethane (NinjaFlex) was used to fabricate the gripper, and thin and flexible fishing lines were used as tendon within the soft fingers.

Angelini’s group demonstrated high quality 3D printing of commercially available silicones by using support materials made of silicone oil emulsion.^208^ They could 3D print an artificial heart valve with a thickness of 250 μm. In another report, Hamidi et al. used multimaterial 3D printing to fabricate starfish shaped soft robots using silicone elastomers. Silicone elastomers were extruded on a heated bed, followed by 3D printing of sugar syrup.^209^ The printed sugar was later dissolved in water to create cavities, which formed pneumatic channels for actuation. The Matusik group demonstrated design and fabrication of complex soft actuators, using multiobject topology optimization and multimaterial drop-on-demand 3D printing.^210^ An actuator consisting of soft and rigid polymers along with a magnetic nanoparticle–polymer composite that responds to a magnetic field was fabricated. The arrangement of the individual actuators is achieved by multiobjective topology optimization while considering the material properties of the material to be used for individual voxels to achieve the desired functional objective. Multimaterial fabrication was also used to create a passive gripper inspired by *Manduca sexta* ^211^ and using the Fin ray effect. The smart design of the gripper involved the intelligent placement of soft and rigid parts to program the deformation to achieve passive and conformal gripping driven by tendons. The gripper was fabricated by Stratasys Connex Objet500 printer with commercial inks and assembled with a few more steps to achieve a fully functioning soft gripper. Sitti’s group demonstrated miniature soft magnetic machines through multimaterial heterogeneous assembly.^212^ They used bottom-up approach to assemble 3D miniature wireless magnetic soft machines at millimeter and submillimeter scale. Through this approach, they could achieve programmable shape morphing, tailored stiffness distribution, negative Poisson’s ratio, directional joint bending, and remagnetization for shape reconfiguration.

In one of the unique demonstrations of multimaterial 3D printing in design of soft robots, Wehner et al. demonstrated a fully functional, untethered octobot robot.^95^ The robot is powered by the catalytic decomposition of hydrogen peroxide on the Pt surface (Figure 4I), which is regulated by the microfluidic logic. The robot was made by both molding and embedded 3D printing techniques. Bartlett et al. fabricated a combustion powered soft robot by multimaterial 3D printing.^213^ The unique design comprised a rigid core and a soft exterior, with stiffness grading spanning 3 orders of magnitude in modulus. Rigid core housed electronics, battery, and controllers, while the soft body functioned as an actuator. Combustion of butane with oxygen powered the soft robot. Experimental and finite elemental analyses of the impact of the falling robot showed that the functionally gradient structures performed better compared to rigid and flexible counterparts. The robot was 3D printed in Stratasys Connex500 3D printer with 9 different layers of robot having modulus between 1 MPa (rubber like material) and 1 GPa (rigid). The direction of motion of the robot was controlled by preinflating one or more of the pneumatic legs of the robot before combustion.

In some soft robotic applications involving operation at a high temperature, the thermal regulation of the actuator is critical. In one such demonstrations, soft robotic actuators are 3D printed using multimaterial SLA consisting of poly-*N*-isopropylacrylamide body coated with microporous polyacrylamide dorsal layer.^214^ The micropores dilate at elevated temperatures (>30 °C) and enable localized perspiration in the actuator (Figure 4J). This sweating enables 600% enhancement in the cooling rate, much better than the cooling capacity of animals. The unique design and choice of materials enable a soft robotic gripper that can both mechanically and thermally manipulate hot objects.

Tauber’s group designed a pneumatic logic gate^215^ to control soft robotic actuators in an attempt to replace conventional electronics-controlled systems. The 3D printed two alternatively acting pneumatic valves can be suitably controlled to perform Boolean operations (OR, AND, or NOT gate) similar to electric circuits. The valves were 3D printed using thermoplastic polyurethanes, Recreus FilaFlex filaments, with different shore hardness from 63 to 82 A. The pneumatic logic gate was used to control a 3D printed robotic walker, a juice dispenser, and exhibited high compliance by being fully functional even when run over by a car. Hubbard et al. realized a fully 3D printed soft robots with integrated fluidic circuitry^216^ that could function similar to a soft robot controlled using electrical components. The key fluidic elements such as fluidic diode and closed and open transistors were realized through multimaterial 3D printing via Stratasys Connex 3D printer. These fluidic valves comprised rigid housing with single stop orifice and multiple bottom orifice, a compliant free floating disc. The floating disc closed/opened the orifices to realize fluid flow or magnified/reduced fluidic pressure. By decisively placing these valves, fluidic circuits could be designed, which can supply different types of inputs, viz., constant pressure, sinusoidal pressure, or varying pressure. Powered by these fluidic circuits, the researchers demonstrated locomotion of a soft robotic turtle that can walk and swim and a soft robotic hand capable of playing a video game. Chen et al. leveraged bistable actuators triggered by SMP for electronics-free propulsion in soft robots.^217^ The actuator, comprising an SMP attached to a bistable element, activated at its glass transition temperature, causing rapid propeller movement and enabling swimming. The soft robot was fabricated in a single step using multimaterial 3D printing with a Stratasys Connex printer utilizing VeroWhite plastic (*T*_g_ = 60 °C) for SMP muscles, Agilus30 elastomer for compliant bistable components, and RGD525 for high-temperature-resistant parts.

### 4.5 Functional Designs

Functional designs are defined by their target function rather than by a specific design principle. They integrate materials and geometries to fulfill that goal and therefore may overlap with other design categories. In one such demonstration, He et al. utilized tubular actuators embedded with LCE to achieve multiple actuation modes, contraction, bending, and expansion.^218^ Using such multiple actuators, a soft gripper and untethered soft robot was fabricated. The wires embedded in the actuators are heated by applying an electric potential, which triggers contraction of the elastomers. By localized control of this Joule heating, various actuation modes were achieved. In another report, a unique approach to soft robot locomotion inspired by plant growth was demonstrated.^219^ This soft robot achieves locomotion through controlled growth, facilitated by 3D printing with thermoplastic filaments. By precisely controlling the amount of material deposited at each location, the direction of growth and thus the robot’s movement can be managed. The extrusion temperature, plotting, and feeding speed of the filaments were carefully regulated to print various geometrical characteristics, enabling the robot to grow and move effectively. Gu et al. developed magnetic soft-robotic chains that self-fold into stable assemblies using combination of elastic and magnetic energies.^220^ These chains, manipulated via a catheter sheath, enable repeated, programmable assembly and disassembly, and are compatible with advanced magnetic navigation for minimally invasive interventions. The chains were fabricated by multimaterial 3D printing of rigid (Verowhite, Stratasys) and elastic (Agilus30, Stratasys) materials. As a proof of concept, they fabricated a tethered capsule endoscope designed from MaSoChains that included camera, magnetic, and biopsy modules, allowing precise navigation and tissue sampling within a stomach model. Heung et al. created an assistive robotic hand with a additive manufatured bidirectional actuator (Figure 4K) for hand-impaired patients’ rehabilitation.^221^ The hand features pneumatically actuated fingers and embedded flex sensors to monitor finger bending angles in real-time. This robotic hand helped patients open and close their fingers and successfully grasp objects with sufficient force for daily activities.

Zhang et al. designed a specialized soft robotic gripper^222^ to capture objects with high kinetic energy. The palm and the bending actuators were made of silicone rubber, and the skeleton was made by 3D printed TPU rubber (Shore 95A) with high elongation (>800%). To enhance the strength of the actuator and the palm Kevlar fibers were embedded within these structures. The gripper works by dissipating and harvesting the kinetic energy of incoming objects within 30 ms and autonomously uses the harvested energy to enhance the grasping force. The gripper was mounted on a drone and could grasp flying objects. Suzomori et al. designed a manta swimming robot,^223^ which is driven by two embedded bending pneumatic rubber actuators. The cross-section shape of the actuator was optimized based on static analysis using a nonlinear finite element method, in which both geometric and material nonlinearity were considered. The actuators and robots were manufactured by a CAD/CAM based rubber molding process. The manta robot could swim at a rate of 100 mm/s smoothly like a manta fish. Tang et al. designed a self-protecting soft fluidic robot inspired by the human hand, featuring sensing and self-healing capabilities (Figure 4L)^224^ The robot actuates using pressure generated by an electrohydrodynamic (EHD) pump. One of the key attributes of EHD pump is that its pumping fluid consists of methyltracetoxysilane (C_6_H_12_O_6_Si) and dibutyltindilaurate (C_32_H_64_O_4_Sn) that promote self-healing. The electrodes of the EHD pump were 3D printed using conductive and nonconductive TPU, actuators were cast with Ecoflex 00–30 silicones, and the E-skin was made of gallium–indium–tin liquid metal alloy embedded in silicone film. In the case of damage to the robot, the E-skin signals the microchip by detecting changes in resistance. When the robot needs repair, the E-skin initiates self-heating, enabling the electrofluid to fill gaps and promote healing. Giordano et al. designed an unmanned underwater vehicle featuring an unconventional soft robotic morphing wing^225^ to achieve a tunable lift-to-drag ratio and adapt to different flow conditions. The morphing wing was composed of two chambers that could be inflated in succession to achieve the desired shape change. The wing was fabricated by using EcoFlex 00-50 from Smooth-On by casting. Actuation was accomplished by using a closed hydraulic cycle driven by a peristaltic pump. Zhai et al. developed a desktop FFF method to 3D print TPU soft robots without electronic components to achieve autonomous grasping.^72^ 3D printing parameters were optimized by having higher extrusion temperature, lower layer height, and increasing the overlap between the layers to achieve airtight pneumatic actuators. By strategic placements of actuators and valves, the grasping and release of the objects was autonomously achieved without dedicated electrical control units.

Interest in soft robots for mental comfort and medical rehabilitation has grown significantly. In one demonstration, Kim et al. designed a soft robotic apparel that provides bilateral assistive hip flexion torques to aid with limb advancement for patients with Parkinson’s disease.^226^ The functional apparel were designed to be worn around waist and thighs and fabricated by 3D printing. The apparel consisted of cable drive actuators and load cells to monitor the force. The gait correction was achieved by delivering the force around the toe-off subphase of the gait cycle. Mohammadi et al. designed a 3D-printed, lightweight (253 g) soft robotic prosthetic hand^227^ with commercial TPU. The hand was actuated using cables and fabricated through monolithic 3D printing of soft materials, incorporating membrane-enclosed flexure joints. This prosthetic hand achieved a power grip of 21.5 N, a finger flexion speed of 1.3 s, and maintained its functionality for over 45,000 cycles. Although not 3D printed, several soft robots were functionally designed for these therapeutic goals. A wearable airbag robot reduced pain and fear through haptic compression,^228^ and a breathing-mimicking sleep robot improved sleep quality and reduced anxiety.^229^

Unlike conventional magnetic soft robots which are driven by external magnetic field, Zhu et al. designed a miniature walking soft robot^230^ inspired by the locomotion posture variation of an inchworm that is powered by its internal electromagnets. The robot consists of Fe_3_O_4_ magnetic particles and PDMS composite surrounding magnetic coils supported on two plastic sheet legs. The electromagnetic coils induce either repulsion or attraction between the adjacent segments of the soft robot, inducing deformation at the joints, which are converted to linear translation by the specially designed plastic legs. The robot achieved precise and stable motion using just 240 mA current at 6 V.

Mao et al. presented a small scale soft electromagnetic robot (SEMRs)^231^ made of curved elastomeric bilayers with embedded printed liquid metal channels. The robot was fabricated by printing liquid metal coils on prestretched elastomer PDMS films. When subjected to static magnetic field, the Lorentz forces acting on the liquid metal carrying alternating current drive the soft robot deformation. By passing a time varying current through the liquid metal coils in the static magnetic field, the desired deformation of the soft robot was achieved. The robot can walk, run, swim, jump, steer, and transport cargo at high speeds in the range of several tens of its body length.

Conventional pneumatically actuated valves require electronics for control and operation. Choe et al. developed a self-sensing tensile valve (STV)^232^ capable of self-sensing and proportional control of soft pneumatic actuators from single constant pressure supply. STV consists of elastomeric inner and outer tubes wrapped within helical yarns and connected to 3D printed connectors at the ends. One of the ends of the connector is attached to the actuator, while the other is connected to the pneumatic source. When STV is not strained (ε = 0) the inner tube is in the open position, resulting in the exit of air through the outlet. In contrast when STV is strained (ε > 0) the stretching of yarns compresses the inner tube, resulting in entry of pressurized air into the actuator and thus inflating it. Thus, pressure inside the actuator is autonomously regulated proportional to the extent of strain in the STV. Taking advantage of this autonomous regulation of pressure, the authors integrated the STV into a self-adaptive exosuit for assistance during lifting actions and an untethered electronics free soft gripper. Bruder et al. designed a soft robot arm capable of lifting heavy weights by introducing localized stiffening at joints without compromising the compliance characteristic of soft robots.^233^ This stiffening was achieved through antagonistic actuation involving two or more actuators acting at a joint with net zero torque. McKibben muscles were used to provide this antagonistic actuation, arranged in a truss pattern around the robot’s spine, enabling joint stiffening when active and allowing free motion when inactive. McKibben actuators have also been applied in implantable extracardiac soft robotic devices for cardiac pumping assistance.^234^ Elastic elements integrated into the soft actuators provide a recoiling function to aid in refilling during the diastolic phase of the cardiac cycle. Interested readers may also refer to in depth reviews^11,235,236^ on various design approaches to soft robots in the literature.

## 5 Sensor Integrated Soft Robotics

Integrating sensors in soft robots is essential for enhancing their functionality, adaptability, and interaction with their surroundings. Sensors provide real-time data on parameters such as pressure, position, temperature, and force, enabling precise decision-making and task execution. Two main types of sensors are used in soft robots: proprioception and exteroception. Proprioception sensors^238^ provide information about the robot’s position in the absolute coordinate system, the relative position of its parts, and the curvature of its deformation during actuation. This information is vital for robotic control to determine input parameters to control the robot through feed-forward or inverse control. Exteroception sensors,^239^ on the other hand, provide information about external stimuli such as force, sight, heat, electric, or magnetic fields, and other relevant environmental parameters. This information is crucial for the robot’s interaction with its surroundings, ensuring appropriate responses to external conditions. Sensors play a pivotal role in understanding the current state of the soft robot and assist in the decision-making process for determining actuation input parameters to achieve the desired goals. The following paragraphs discuss various examples of sensor integration in soft robots, and key examples are presented in Figure 5.

**Figure 5:**
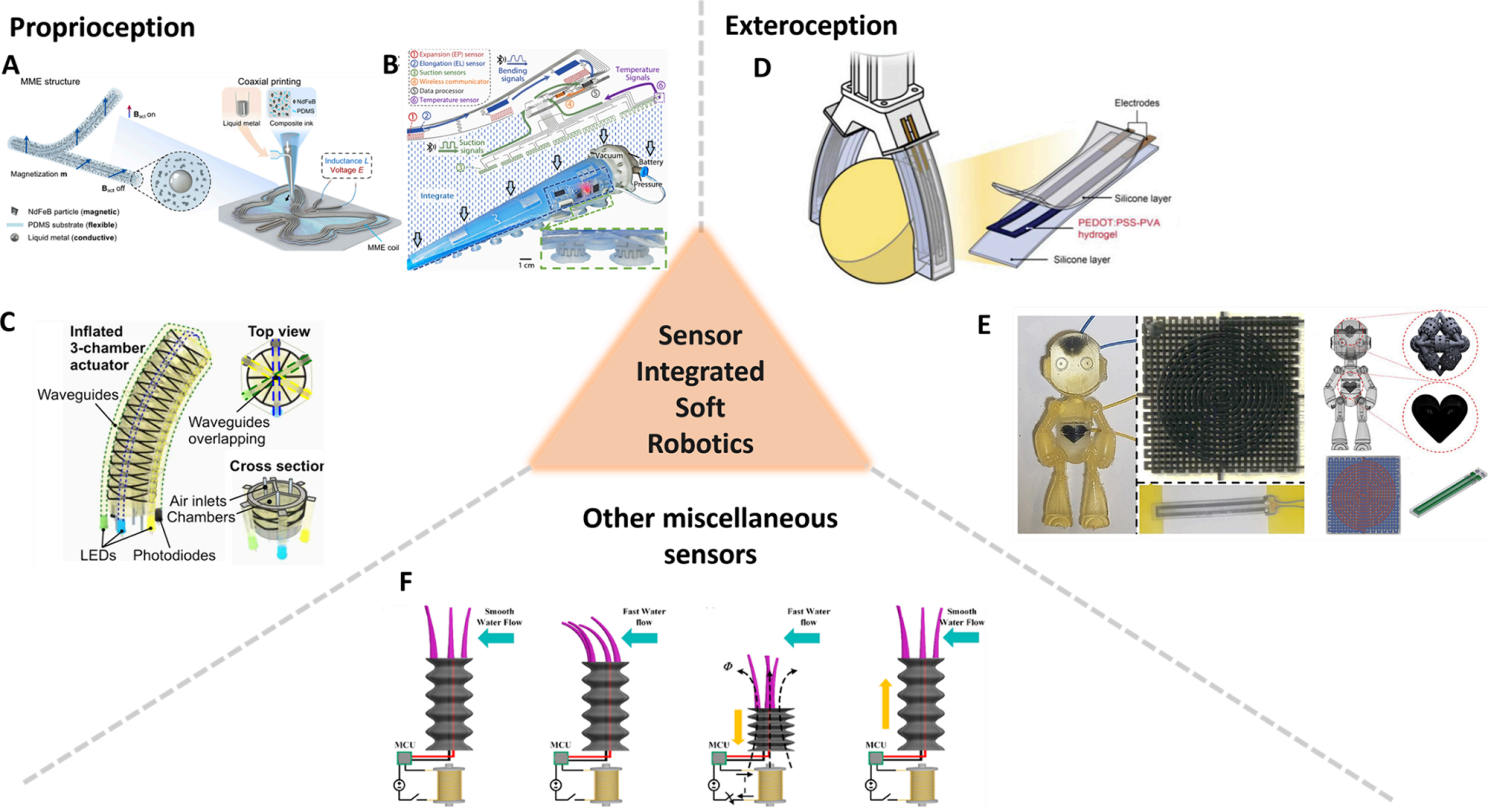
Sensing in soft robotics. Proprioception. (A) Magnetic mechanical electrical hybrid soft robot with actuation and sensing functionality. The soft robots are 3D printed using coaxial extrusion of a liquid metal core and magnetic silicone composite sheath. Reproduced with permission from ref 240. Copyright 2023 Springer Nature under Creative Commons Attribution 4.0 International License 4.0 (CC BY). (B) Octopus inspired sensorized arm. The liquid metal sensors embedded within the octopus inspired arm sense bending, suction, and temperature, process the signal using on-board electronics, and transmit the data wirelessly for closed loop feedback. Reproduced with permission from ref 242. Copyright 2023 American Association for the Advancement of Science. (C) Design of 3D printed biogel based multidirectional soft actuator with embedded optical fibers. Three optical fibers are integrated within the omnidirectional actuator to provide information related to bending and normal force at the tip. Reproduced with permission from ref 246. Copyright 2022 American Association for the Advancement of Science. Exteroception. (D) Highly stretchable hydrogel strain sensor fabricated by 3D printing and freeze–thawing enables physiological signal monitoring and object recognition in soft grippers. Reproduced with permission from ref 256. Copyright 2022 Wiley-VCH, under Creative Commons Attribution 4.0 International License (CC BY). (E) Multimaterial resistive sensor and soft gripper 3D printed in a single step using Stratasys Connex3 printer for object size estimation via strain sensing. Reproduced with permission from ref 262. Copyright 2019 Frontiers, under Creative Commons Attribution 4.0 International License (CC BY). Other miscellanous sensors. (F) Sensing of flow and self-protection of the sea-anemone inspired soft robot in the presence of fast water flow. The magnetic soft robot consists of tentacles which measure the water velocity through generated electrical signal, which triggers the magnetic coil to compress the soft robot to stop being swept away in the current. Reproduced with permission from ref 280. Copyright 2021 Wiley-VCH, under Creative Commons Attribution 4.0 International License 4.0 (CC BY).

Sensors for proprioception. Zhang et al. fabricated hybrid magnetic–mechanical–electrical (MME) core sheath fibers (Figure 5A)^240^ through coaxial 3D printing of liquid metal (Ga_3_In) core and soft magnetoactive (NdFeB nanoparticles in PDMS) composite sheath as the shell. This core–shell composite enabled excellent electrical conductivity, robust mechanical properties, and durability to 3D printed structures. These composites were used to make a fiber catheter to perform minimally invasive electroablation surgery and a somatosensory soft gripper that can identify and sort objects. Li et al. developed a robust, power-free biohybrid mechanoluminescent soft robot by encapsulating bioluminescent dinoflagellates in elastomeric chambers.^241^ The robot was created by injecting dinoflagellate solution into silicone elastomer chambers and sealing the holes with silicone. Dinoflagellates, sensitive to mechanical strain, produced mechanoluminescence to visualize external perturbations and deformations under low-light conditions. The biohybrid system maintained its functionality for several weeks.

Xie et al. fabricated an electronics-integrated soft octopus arm (E-SOAM) for advanced environmental interaction involving reaching, sensing, and grasping in a large domain (Figure 5B).^242^ The octopus arm and suckers were created by casting silicone elastomers into 3D printed molds. The liquid metal electronics circuit and sensors were embedded in silicone by using a transfer method, followed by the placement of IC chips and other electronic components. The entire device was then encapsulated within a silicone film to achieve fully embedded electronics. E-SOAM used a bending–elongation propagation model to move, reach, and grasp the aluminate efficiently. The electronic circuitry, capable of withstanding 710% stretching due to the use of liquid metals, allowed E-SOAM to process bending, suction, and temperature sensor information, even under significant deformation. As a result, E-SOAM could mimic the grasping abilities of an actual octopus, performing reaching, grasping, and withdrawing motions up to 1.5 times its original arm length. Liquid metal-based sensors have also been reported for detecting strain and pressure variation within earthworm^243^ inspired soft robots that can travel inside pipes and difficult to reach corners. Yang et al. demonstrated proprioceptive sensing in soft robots using multifunctional conductive polymer strings^244^ for both actuation and sensing. Supercoiled polymer artificial muscle strings (SCPAM) made from conductive nylon sewing threads were used to actuate the robot. Changes in resistance due to the extension of these strings provided proprioceptive feedback. Similarly, Farrow et al. used liquid metal embedded soft gripper with a pressure sensor^245^ to map the curvature of the soft robot based on air pressure and the resistance of the liquid metal strain sensor.

Kaltenbrunner’s group demonstrated 3D printing of biodegradable resilient biogels^246^ as omnidirectional and exteroceptive soft actuators (Figure 5C). Fully degradable biogel was made of mixture of glucose, glycerol, gelatin, and citric acid in water and 3D printed using thermally controlled extrusion 3D printing. Highly stretchable (>600%) biogel was 3D printed in the form of pneumatic actuators for omnidirectional movement with fast response (<1 s) with integrated waveguides for both proprioception and exteroception. The actuator was used for dynamic control of search and wipe routines to detect and remove obstacles. Zhao et al. embedded stretchable optical waveguides within 3D printed soft grippers for proprioception and force sensing.^247^ They correlated optical losses due to bending and compression to bending and contact force to achieve sensing. The smart soft gripper could estimate surface roughness, object size, and softness, demonstrating capabilities similar to those of a real hand. The stretchable optical fibers were made by casting transparent polyurethane rubber (VytaFlex 20, Smooth-on) as a core inside Elastosil (M 4601 Wacker Chemie AG) clad. Van Meerbeek et al. presented an internally illuminated elastomer foam that can detect its deformation pattern through analyzing the sensor data using machine learning models.^248,249^ Multiple optical fibers were used within the foam and the diffused light from the optical fibers were interpreted to predict the foam’s motion such as clockwise, counterclockwise, upward bending, or downward bending motions to address the nonlinear time behavior of soft materials in sensors and actuators.

Ozel et al. reported a noncontact measurement of the curvature of a soft robot by utilizing a magnet and an electronic Hall effect sensor.^250^ Sensor accurately mapped the curvatures with a room mean square error of 0.023 cm^–1^ at 7.5 Hz under both static and dynamic conditions. Ha et al. developed reconfigurable magnetic origami actuators^251^ with proprioception. These actuators, made of shape-memory polymer (DiAPLEX, SMP Technologies) films, embedded with magnetically aligned NdFeB microparticles, bend and fold when exposed to external magnetic fields and photothermal heating. The folding location was controlled by selectively illuminating specific areas of the actuator. Additionally, high-performance magnetic field sensors on 3 μm thick Mylar foils were laminated onto the origami actuators. These sensors detected in-plane magnetic fields using the giant magnetoresistance (GMR) effect and out-of-plane magnetic fields using anomalous Hall effect sensors. This allowed the origami actuators to assess both the state of the magnetic actuator and the external magnetic field to guide their actuation.

Zhou et al. integrated a solenoid-shaped liquid metal sensor onto snakelike soft robots to precisely detect both tensile and bending deformations.^252^ The sensor was fabricated using coaxial coprinting of liquid metal and silicone rubber. It was employed to measure the curvature of a finger and provide positional feedback for an endoscope. Yang et al. developed a low-cost soft gripper with paper electronics^253^ that endowed with shape and proximity sensing abilities. Paper based resistive strain sensors (RSS) and capacitive proximity sensors (CPS) were fabricated by printing nanosilver ink (NBSIJ-MU01, Mitsubishi) on resin coated papers by using a general-purpose commercial inkjet printer (MG7530, Canon). The RSS exhibited low hysteresis (0.01%) and detected bending angle of the gripper and hence estimated the size of the objects grasped. The CPS detected object proximity within 8 mm and differentiated objects by their permittivity.

### 5.1 Sensors for Exteroception

Due to ongoing demand for automation, there have been intense efforts in the field of soft robotic grippers for automation in various fields. Shibo et al. demonstrated control of the soft grippers without embedding dedicated sensors by monitoring the pressure inside the soft gripper (using pressure connected remotely) during actuation and its interaction with the environment. The sensing mechanism depends on the change of internal volume which results in pressure variation within the soft gripper.^254^ The versatile technique was used to sense roughness, shape, size, and stiffness of the objects and could be retrofitted on a variety of suction and positive pressure actuated soft grippers. Yang et al. measured the contact force and bending curvature of a soft gripper using a pneumatic soft sensor (PSS)^255^ integrated within the gripper’s pneumatic chamber. The sensor monitored pressure changes in relation to the gripper’s bending and contact force.

Shen et al. developed highly stretchable, ultralow-hysteresis conductive polymer hydrogel^256^ strain sensors for soft robots (Figure 5D). The sensor, made of a microphase-separated network of poly­(3,4-ethylenedioxythiophene):polystyrenesulfonate (PEDOT:PSS) nanofibers and poly­(vinyl alcohol), was fabricated by 3D printing followed by freeze–thawing. It could measure strains over 300% with low hysteresis (<1.5%). When integrated into a soft gripper, the sensor enabled measurements of physiological signals, hand gestures, object recognition, and remote control of the robot. Yamaguchi et al. integrated a 2 × 2 array of tactile force sensors into a soft robotic hand to detect object slipping by measuring the time delay of tactile forces.^257^ This information enabled real-time control of slip-free grasping. The resistive tactile sensors were made by coating single-walled carbon nanotubes (SWCNT) on paper and encapsulating them in silicone. Bilodeau et al. used liquid metal strain sensors in soft robotic grippers to detect object grasping.^258^ The sensors showed a clear change in resistance when the gripper contacted an object compared to when it was actuated without contact. Zhang et al. used a triboelectric sensor^259^ for nondestructive sorting of objects based on their size. Truby et al. employed embedded 3D printing to create soft robotic fingers with discrete actuation modes and integrated ionogel soft sensors^260^ for proprioceptive and tactile sensing. The ionogel, composed of 1-ethyl-3-methyl-imidazolium ethyl sulfate with 6 wt % fumed silica particles, was embedded within a silicone elastomer actuator using embedded 3D printing. These tactile sensors enabled autonomous object grasping by detecting distinct resistance changes for each mode of grasping.

Shih et al. used data from flexible sensory skins^261^ and an analytical model of a soft gripper to construct 2D and 3D models of objects. The soft gripper had three pneumatically actuated channels, each embedded with a resistive sensor made of a 12% MWCNT-PDMS composite with ∼6 S/m conductivity. These sensors measured both the bending of the actuator and the force at the fingertip. In another work, Shih et al. designed and 3D printed a multimaterial resistive sensor^262^ for soft robots to estimate object size during grasping (Figure 5E), through strain sensing. The gripper and sensor were fabricated in a single step using Stratasys resins on a Connex3 Objet350 printer.

Yin et al. used a combination of proximity, pressure, and orientation sensors within a soft gripper to detect and respond to external disturbances that can interfere with the grasp, release, and transport of objects.^263^ Chen et al. incorporated triboelectric nanogenerator (TENG) sensors into soft pneumatic actuators.^264^ TENG consisted of nickel sponge electrodes for its ease of integration within soft chambers. The TENG sensors accurately correlated output voltage with bending angle, facilitating smart gripping and object size/weight estimation. Jin et al. utilized a TENG sensor in soft robotics for digital twin applications.^265^ Their TENG sensor detected contact position and area, while a gear-based sensor estimated the degree of elongation in the soft gripper. These data were trained using a support vector machine algorithm, achieving the identification of diverse objects with 98.1% accuracy.

Justus et al. fabricated a hybrid bio-LED-actuator soft gripper capable of detecting chemical signals in the environment.^266^ The gripper used engineered *Escherichia coli* bacteria to detect chemical signals and a flexible light-emitting diode (LED) circuit to convert biological signals into an electronic signal. It was employed for pick-and-place operations of objects from a bath. When immersed, the sensors searched for chemical signals and relayed the information to the robot, which then decided whether to continue the operations or alert the operator about the chemical presence.

In another report, Hegde et al. designed a metamaterial based optical sensor^267^ to be integrated within soft robotic grippers for force sensing. The range and sensitivity of the sensor could be programmed by the choice of material properties and dimensions of the metamaterial lattices. The sensor was integrated within a variety of soft grippers,^268^ and tactile feedback was used for closed loop control of grasping for food tray assembly. Larson et al. developed a highly stretchable electroluminescent (EL) skin^269^ with tactile sensing capabilities. They created EL films by layering a ZnS phosphor-doped dielectric elastomer between transparent hydrogel electrodes composed of a polyacrylamide–LiCl composite. Changes in the luminescence and capacitance of these EL sheets occurred with deformation. When they were integrated into soft robots, these changes in color and capacitance provided crucial feedback on external and internal stimuli.

Zou et al. reported a soft robotic gripper with an intrinsically embedded pressure–temperature sensor.^270^ The pressure sensor was made of an MWCNT/PDMS composite, while the temperature sensor was fabricated using a carbon black–PDMS composite. Pressure changes were measured by capacitance changes at the electrodes, and temperature changes were measured by resistance changes. The dual-mode sensor had a vertically stacked bimodal configuration, which separated the two sensing elements to avoid signal interference. The sensor enabled the soft robot to perceive the size, temperature variations, hardness, and weight of objects, supporting advanced robotics control. Yang et al. reported a multifunctional soft robotic finger integrated with temperature and pressure sensors for detecting various classes of materials during pick-and-place operations.^271^ The nanowire-based temperature sensor was made of PEDOT/PSS nanowires fabricated through a nanocapillary filling method. As the temperature increased, water molecules were released from the hydrophilic PSS shell, causing the hydrophobic conductive PEDOT core to come into closer contact, resulting in a reduction in the resistance. The conductive sponge pressure sensor was created by immersing a polyurethane sponge in a PEDOT/PSS solution for 15 min, then squeezing out the excess solution to produce a conductive sponge. The electrical resistance of the sponge decreased with increasing pressure due to the increased contact surfaces, which was used to measure pressure.

Qiu et al. reported on a biomimetic Drosera Capensis^272^ with multimodal sensing and self-regulated actuating capability through closed loop control of sensing and actuating system. The soft robot incorporates a thermally responsive actuator equipped with a programmable, flexible heater. A PEDOT:PSS layer serves as the thermally responsive element atop a thermally inert polyimide layer, inducing bending of the actuator due to differential elongation. Additionally, the actuator was coated with a piezoelectric layer (patterned liquid metal) and a piezoelectric sensor (poly­(vinylidene fluoride)–trifluoroethylene, P­(VDF-TrFE)) to detect physical interactions with the surroundings. The sensor layer triggers Joule heating via the embedded flexible heater, inducing bending in response to thermal stimuli, thereby achieving self-regulated actuation.

### 5.2 Other Miscellaneous Sensors

Kim et al. devised a sustainable method^273^ for sensor manufacturing in soft systems using self-coagulating conductive pickering emulsions. Unlike conventional solvent-based printing methods that are prone to swelling and decomposition, their approach utilized ethanol-based emulsions that spontaneously coagulate into conductive composites. The system employed PDMS precursors stabilized with conductive nanoparticles in ethanol, forming emulsions that polymerized upon ethanol evaporation and atmospheric moisture contact. This innovative ink sensitized soft robots and textiles with high strain sensitivity and minimal hysteresis.

Rentschler’s group has demonstrated a soft robotic multifunctional shape display^274^ that can shape morph at high frequencies (50 Hz), senses deformation within 0.1 mm and force sensitivity of 50 mN. The soft display was driven by an array of 10 × 10 hydraulically amplified self-healing electrostatic (HASEL) actuator. An interference free magnetic sensor was embedded directly in the surface layer to sense the deformation and force. The soft robot was used for user interaction, dynamic manipulation of both liquids and solids, displaying images, and measuring the mass of objects. Byun et al. designed an electronic soft skin^275^ for wirelessly activated soft robots. The skin, consisting of 82 surface-mount devices (SMDs), was assembled with Ag epoxy interconnects and encapsulated in a silicone elastomer. This conformable e-skin enabled wireless communication for controlling the soft robot.

Wang et al. designed a differential soft sensor^276^ for estimating the interactive force and assistive torque in a robotic hip exoskeleton. The force sensor was made of soft air chambers made of thermoplastic polyurethane. The differential pressure sensors monitor the pressure within these soft air chambers to estimate the interaction force between the thighs and the exoskeleton. Zhao et al. embedded engraved optical fibers within a soft robotic exoskeleton to enhance force augmentation and feedback control.^277^ The loss in optical signal due to bending and interaction forces between the user and the exoskeleton was used for closed-loop control in soft orthosis. Gu et al. designed a soft neuroprosthetic hand^278^ that can provide simultaneous myoelectric control and tactile feedback weighing only 292 g. It featured six active degrees of freedom under pneumatic actuation and was controlled by four electromyography sensors. The hand included five elastomeric capacitive sensors on the fingertips for tactile feedback. These sensors were made of ionic gel with polyacrylamide hydrogel containing a LiCl salt, which ensured conductivity and moisture retention. The soft neuroprosthetic hand outperformed conventional rigid prosthetic hands in speed and dexterity. An individual with transradial amputation was able to regain primitive touch sensation and achieve real-time closed-loop control. Yeo et al. integrated a strain sensor into a soft robotic rehabilitation glove^279^ to monitor finger kinematics. The glove, made from soft silicone (DragonSkin 10, Smooth-on), featured a strain sensor comprising a screen-printed silver nanoink on a silicone elastomer substrate. This sensor detected deformations exceeding 20% with a high gauge factor of 50,000, enabling detection of irregular finger movements and assessment of finger stiffness and dexterity.

Wang et al. designed a sea anemone inspired soft robot (Figure 5F)^280^ that can sense water velocity and take actions for its protection. The robot was made of a NdFeB/silicone magnetic composite. In the event of high-water velocity, the tentacles of the soft robot converted the mechanical stimuli to electrical signals and triggered the robot to deform to avoid being swept away in the water. Wall et al. introduced an acoustic sensing approach to measure the location, inflation, contact force, and surrounding temperature of actuators.^281^ The sensor operates on the principle that sound modulation occurs as it travels through the actuator, depending on its state (e.g., shape and contact force). By detecting small changes in the sound’s frequency pattern and feeding this data to a machine learning model, the exact state of the actuator could be estimated. Additionally, the rubber of the actuator shielded against background noise that could potentially interfere with measurements. This sensing method utilized a MEMS condenser microphone (Adafruit SPW2430) and a balanced armature speaker (Knowles RAB-32, 063-000) embedded within a PneuFlex actuator made of silicone rubber. Several other mechanisms^363^ and integration of the sensors in soft robots have been described in our previous reviews^238,282^ and other reports.^283–287^

## 6 Applications of Soft Robotics

Soft robotics fabricated through additive manufacturing offers unique advantages of compliance, versatility, and adaptability over traditional rigid robots.^288–290^ Their ability to deform, conform to complex shapes, and interact safely with humans and delicate environments has opened up new application opportunities across numerous fields, as discussed throughout this review. In this section, a comprehensive bibliographic analysis of the applications of soft robots is presented. To conduct the study, we first identified keywords associated with notable applications of soft robots from highly cited reviews and papers. These keywords helped categorize the literature into four main fields: medical, locomotion, wearables, and manipulation, which represent the core areas where soft robots are applied. Then, to gather relevant literature, we performed searches in databases by combining the term “soft robotics” with each application search keyword. Finally, we arranged the data and neglected irrelevant papers and duplicates arising from multiple application topics to avoid double-counting. It should be noted that duplicates from different main fields have been counted twice since the robot can be used for various applications. The bibliographic review, pertinent to the period during which the manuscript was prepared (April 2024), reflects the overall trends and indicates potential future developments within the field. The bibliographic review is presented in Figure 6.

**Figure 6:**
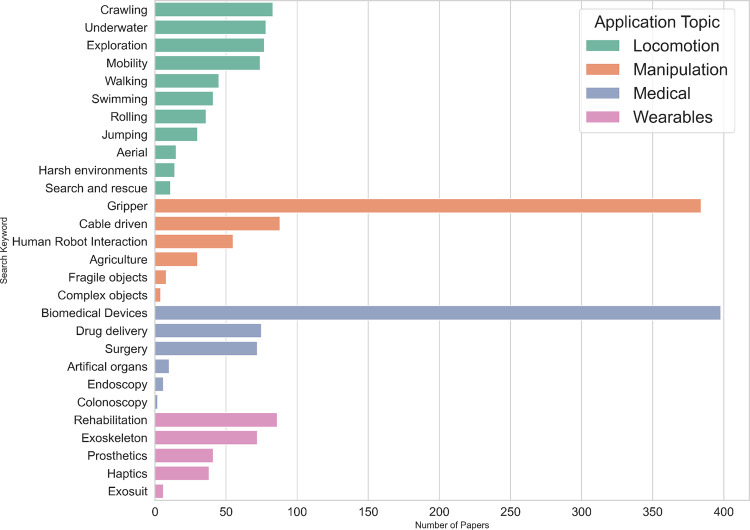
Bibliographic analysis of soft robotics applications. Number of publications by search keyword, grouped by application topic. Four main domains are highlighted: locomotion (blue), manipulation (orange), medical (green), and wearables (red), illustrating the relative research focus across different application areas.

As shown, the research reports are mainly in four fields of application: medical, manipulation, locomotion, and wearables. The medical field dominates with 563 total publications, driven primarily by research in biomedical devices (398 publications), drug delivery (75 publications), and surgery (72 publications). The manipulation field follows with 569 total publications, focused mainly on soft grippers (384 publications). In the locomotion field, with 504 total publications, research is spread on various types of locomotion, such as aerial (15 publications), walking (45 publications), rolling (36 publications), and underwater applications (119 publications). And finally, the wearable field has 243 total publications, primarily investigating rehabilitation (86 publications) and exoskeletons (95 publications) devices, general wearable applications such as haptic interfaces (38 publications), and in addition devices that could not be categorized (76 publications).

Across all fields, the most extensively researched areas are biomedical applications and grippers for manipulation. In contrast, subtopics like flying or swimming soft robots and certain wearable applications such as exosuits have received less attention due to the challenges in developing suitable materials and designs that can meet the high functionality requirements of these domains. The following sections provide a detailed exploration of each application field, highlighting representative examples, current challenges, and future research directions.

### 6.1 Soft Robotics for Medical Applications

Unlike their rigid counterparts, soft robotics can absorb impact energy and deform, reducing potential hazards, particularly in the medical field where the devices are closer to humans.^291,292^ This compliance is crucial in domains such as surgical interventions^291^ and biomedical devices.^293^ Furthermore, advancements in soft robotics fabrication, mainly through 3D printing, enable limitless design possibilities for soft robots. This enabled the development of medical devices for surgery, drug delivery, and artificial organs that potentially surpass the capabilities of rigid robots.^18^

#### 6.1.1 Soft Robotics Devices for Surgery and Endoscopy

In recent years, advancements in surgical tools and assistive robots have made surgery safer and more accessible by reducing the risk of injury and human error. These systems offer numerous advantages, overcoming many limitations associated with minimal-invasive surgery (MIS). They enhance dexterity, restore hand-eye coordination, provide ergonomic benefits, and improve visualization. Furthermore, these technological advancements have made complex or unfeasible surgeries now possible.^291^ However, despite the advancements brought by these technologies, they still predominantly rely on rigid tools, posing challenges in flexibility and access to complex surgical targets. Soft robots have shown great potential for the next generation of instruments due to their inherent compliance and ability to conform to the surrounding anatomy. Several types of soft robotic devices relying on three mechanisms have been developed for various surgical applications:^294^

##### Continuum Soft Robots

These are robots that are able to bend continuously, thus can typically achieve large bending angles.^292^ This type of robot is suitable for MIS or endoscopic procedures because they require only one entry point and the ability to navigate through tortuous paths in the body. For example, Chauhan et al. 3D printed and assembled a silicone (Dragon Skin 10, Smooth-ON) pneumatic origami-inspired 3-channel monolithic soft robotic actuator designed for upper gastrointestinal endoscopic applications,^295^ with 8.5 mm diameter multichannel structure, scalability, and a central hollow channel for an endoscopic camera or tool. The actuator shows high bending efficiency, low radial expansion, and the ability to incorporate an endoscopic camera. In addition, Joe et al.^204^ reported a fully 3D printed jointless continuum soft robot design by graduated stiffness along the actuator made by a porous PUA, achieving 2 degrees of freedom with a high bending angle by using a single pneumatic source. This actuator microporousity made it suitable for MIS because it will not harm organic tissue when maneuvering over it.

##### Peristaltic Robots

Peristaltic robots are self-propelled devices that depend on anisotropic friction to achieve locomotion; for example, an inchworm-like robotic is used for colonoscopy. The robot movement is achieved by using balloons for anchoring the intestines and a rubber spring and cables for crawling by elongation and contraction. The authors showed successful upward movement through pig intestines and showing the potential use for different colonoscopy procedures such as biopsy, water jetting, etc.^296^

##### Serial Robots

Serial robots consist of several prismatic or rotational joints that are coupled together by links. Although not directly fabricated by additive manufacturing, the concept is well demonstrated by Russo et al.^297^ In their work, a soft pneumatic multiarticulated soft robotic arm, made of biocompatible silicone elastomers such as MED4-4220 and MED-6033, was based on a pop-up book microelectromechanical systems (MEMS) manufacturing method. The fabricated arm is integrated on a flexible endoscope and showed the ability to perform tissue contraction in vivo.

#### 6.1.2 Soft Robotics for Drug Delivery

Soft robots have great potential for targeted and controlled drug delivery in the human body because they offer a promising solution to some of the challenges faced in traditional methods, particularly when targeting remote or delicate areas. They can be fabricated from soft, biocompatible materials and can be 3D printed with limitless design possibilities,^7,18^ including submillimeter sizes.^293,298^ This enables them to navigate to delicate areas in the body and activate them there for precise drug delivery. For this category of application, the typical actuation concept is based on stimuli-responsive materials^299–301^ which release a drug in response to heat or light. For example, Cabanach et al. developed stealth microrobots via two-photon polymerization using zwitterionic hydrogel photoresists based on carboxybetaine and sulfobetaine, enabling the fabrication of biocompatible, magnetically actuated 3D microrobots (∼20 μm length) capable of encapsulating therapeutic agents for controlled drug release while avoiding immune recognition for over 90 h in vitro.^302^

Additionally, Keneth et al. developed a 3D printable soft two-lid-box based on a shape memory polymer, which opens and closes in response to heat. Moreover, to demonstrate the potential for drug delivery in the body where heat cannot be applied externally, they showed that by combining photothermal materials such as CNT, they can control the box opening state by exposing it to UV.^299^ Similarly, Berger et al. fabricated a soft thermomagnetic microgripper. The microgripper is made by embedding magnetic iron oxide nanoparticles into a soft network of hydrogel and polypropylene fumarate.^301^ They demonstrated the possibility of untethered guide of the gripper using a magnetic field and then controlling its opening and closing state at body temperature.

Another interesting approach involves incorporating artificial intelligence (AI) and soft robotics into an implantable drug delivery release device, such as the FibroSensing Dynamic Soft Reservoir (FSDSR).^303^ This device allows for consistent medication release by bypassing issues caused by scar tissue formation. The FSDSR can sense fibrotic capsule formation and use AI to change the shape by inflation, ensuring consistent drug dosing despite fibrosis. This integration of AI and soft robotics advances the potential of implantable devices to provide long-lasting therapeutic action.

#### 6.1.3 Soft Robotics as Artificial/Augmented Organs

Artificial organs are devices made from active materials that replicate the physiological function of the body. Augmented organs are enhanced or artificially modified body parts designed to improve or restore their natural function through technology.^304^ As implants, they require high standards of functionality, biocompatibility, and specific designs that will limit the fibrotic response of the human body. Soft robots, as active soft materials, can be fabricated to meet these requirements, providing significant advancement in this field.^293^ Such soft robotics devices include artificial blood pumps,^234,305–307^ diaphragm muscles,^192,308^ and excretory devices.^309,310^ Chors et al.^307^ conducted a study on a pneumatically driven artificial heart fabricated by casting commercial silicone in a 3D-printed mold to create a heart as a single monoblock. Their design incorporates three elastomeric chambers: two ventricles (left and right) and an expansion chamber. By inflating the expansion chamber using an external pump, the two ventricles are squeezed, causing the displacement of blood and resulting in a pulsatile flow. They showed that they can recreate heart movements and create a physiological blood flow. However, the artificial heart did not function for more than 3000 beats due to material durability. More specific approaches for using soft robotics have been shown by fabricating actuators to restore the ejection capabilities of falling hearts. For example, pneumatic artificial muscles (PAMs) were used as implants around a heart to support its damaged activity^6,234,311^ or more invasive intercardiac devices to augment the blood ejection.^306^

Similar to the heart’s pumping action, the diaphragm, a vital respiratory muscle, contracts and relaxes in a coordinated motion for breathing. This simple mechanism enables the fabrication of soft robot devices that mimic or aid the diaphragm movement. For example, Hu et al. developed a PAM actuator implanted above a failing diaphragm to mechanically augment its function during inhalation.^308^ Finally, muscles that control the openings and closings of body passages and orifices can also be replaced by soft robots due to their simple valve actuation. Bliah et al. developed by 3D printing a valve actuator from a soft polyurethane foam, and they demonstrated that the valve opening state could be controlled at low pressures of 0–10 kPa.^75^

### 6.2 Wearable Soft Robotics

Wearable robots are devices designed to assist, enhance, and augment various aspects of human functionality, ranging from movement assistance to sensing, and communication. These systems are typically categorized as either devices that provide physical actuation, such as exoskeletons and soft exosuits, or devices that facilitate interaction through sensing and feedback, such as haptic interfaces.^312,313^ While electronic skins (e-skins) also play a vital role in wearable systems by enabling tactile sensing,^314,315^ they are considered auxiliary components rather than standalone soft robots, as they do not actively deform or produce motion. Soft robotics offers advantages over rigid robotics in wearable applications because it provides lightweight, soft, and adaptable devices. This flexibility allows for more natural interaction with the wearer’s body, enhancing comfort and functionality and enabling the fabrication of more advanced soft wearable robots, which is discussed in the following subsections.

#### 6.2.1 Soft Prosthetic Robots

The integration of soft robotics in wearable prosthetics represents a significant advancement in enhancing user experience through improved adaptability, comfort, and functionality.^293,316^.^317^ Soft robotic prosthetic limbs can better conform to various objects, providing more natural and dexterous interactions,^7^.^318^ The soft materials used, such as textile,^319^ silicone,^316^ and TPU,^320^ offer a more comfortable and natural feel, promoting better integration and acceptance by the user while reducing the risk of injury from impacts or pinching,^316^.^321^ Moreover, the inherent compliance of these materials allows for more intuitive control, potentially facilitating learning and skill acquisition.^316^ Furthermore, advancement in 3D printing of soft robotics allows the precise fabrication of complex, bioinspired structures.^3^ This capability enables the creation of customized prosthetic components that can perfectly match the unique anatomical and functional needs of each individual user, enhancing both comfort and performance.^322^ For instance, Caspi-Morales et al.,^316^ compared in their study the use of rigid and soft poly articulated prosthetic hands in nonexpert myoelectric users. Participants underwent training and testing sessions with both types of prosthetic hands to assess the embodiment, functionality, and user experience. It was demonstrated that soft poly articulated prosthetic hands showed advantages in terms of embodiment, multitasking capability, and user experience compared to rigid prosthetic hands. Although this study only highlights the exceptional advantages of soft materials, similar experiments with fully functional soft robots are still required. A first step toward this goal has been demonstrated with a fully 3D-printed soft robotic prosthetic hand with multiarticulating capabilities made by commercial TPU. The fingers are actuated using a cable-driven system. Each finger, including the thumb, index, middle, ring, and little, has multiple joints (distal, proximal, and metacarpal) that allow for independent movement. Various grasp types, such as pinch, power, and tripod grips are shown in Figure 7 in the hand representative image.^320^ Despite these advancements, the development of large, fully functional soft robotic limbs and prosthetics is still in its early stages and there is significant room for further research.

**Figure 7:**
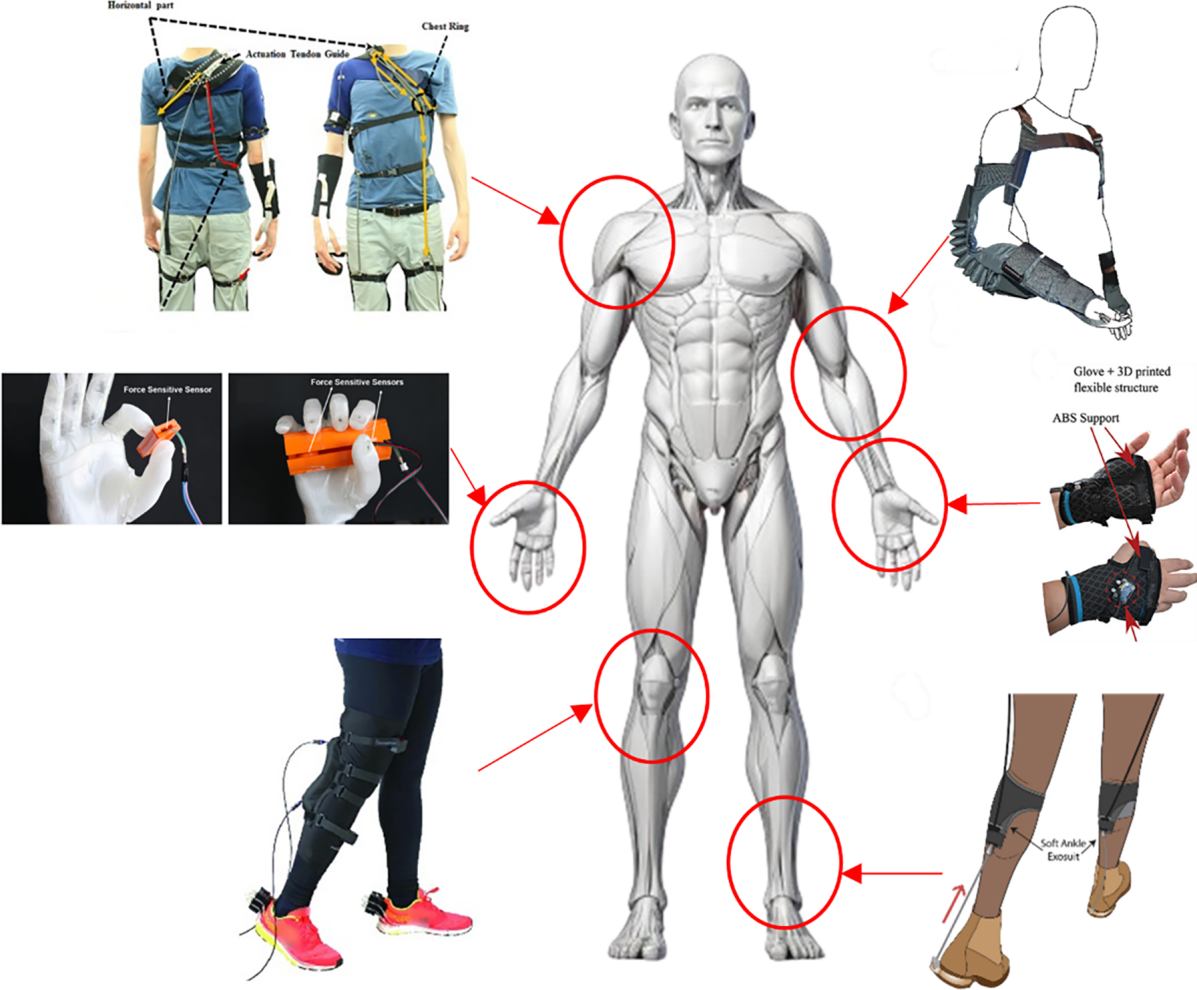
Schematic illustration of the application of soft robotics exoskeletons and prosthetics devices for almost every joint in the body including elbow. (A) Reproduced with permission from ref 346. Copyright 2021 Springer Nature, under Creative Commons Attribution 4.0 International License 4.0 (CC BY). (B) Wrist. Reproduced with permission from ref 348. Copyright 2020 Frontiers, under Creative Commons Attribution 4.0 International License 4.0 (CC BY). (F) Ankle. Reproduced with permission rom ref 353. Copyright 2021 reproduced with permission from AAAS. (C) Knee. Reproduced with permission from ref 331. Copyright 2018 Frontiers, under Creative Commons Attribution 4.0 International License 4.0 (CC BY). (E) Prosthetic hand. Reproduced with permission from ref 320. Copyright 2020 PLOS One, under Creative Commons Attribution 4.0 International License 4.0 (CC BY). (D) Shoulder. Reproduced with permission from ref 328. Copyright 2020, PLOS One, under Creative Commons Attribution 4.0 International License 4.0 (CC BY).

#### 6.2.2 Soft Exoskeletons and Rehabilitation Devices

Exoskeletons and exosuits are wearable robots designed specifically for movement assistance. While traditional rigid exoskeletons offer substantial load transmission and joint support, they often face challenges such as large size, weight, and misalignment with human joints, leading to decreased comfort and increased metabolic cost. Soft robotics overcomes these challenges using compliant materials,^6,7,14,323^ which mimic muscles and tendons for lightweight and adaptable support.^5,312,321^ Nowadays, researchers have developed soft wearable robots for practically every joint in the body, including shoulder,^319,324–328^ knee,^329–332^ hip,^333–338^ elbow,^339–347^ wrist,^348–351^ ankle^352–356^ and more, as demonstrated in Figure 7, offering versatile solutions for a wide range of mobility needs.

Common actuation mechanisms in wearable soft robots include pneumatic and cable-driven systems which are designed to assist muscles to move joints effectively, allowing or aiding to its movements.^5,312^ To showcase this, O’Neil et al.^319^ have investigated the actuation mechanics of unfolding textile-based pneumatic actuators for joint support to present how the design geometric parameters influence the generated moment. Through experimental characterization, three performance regimes (ahearing, creasing, and flattening) were identified, each exhibiting distinct behaviors based on actuator angles and pressures. Their study offers a foundation for designing more predictable and effective textile-based actuators for wearable soft exoskeletons.

#### 6.2.3 Soft Haptics Devices

Haptics, known as “the science of touch”, involves sensing and feedback mechanisms through kinesthetic and cutaneous receptors, enabling tactile human–machine interactions.^357^ Increasing interest in haptic technology, driven by its applications in robotics, virtual/augmented reality, and healthcare, has led to the development of various tactile feedback devices. Many still rely on rigid components that hinder fabrication complexity and natural tactile feedback. Soft haptic devices, however, utilize compliant materials and the actuation principles of soft robotics to achieve the desired sensory experience. Most of these devices use volumetric changes for haptic responses, primarily achieved through pneumatic actuation.^357–359^ For example, Yoshida et al. developed a 3-DoF soft pneumatic haptic device, which provides multimodal feedback to the forearm, integrating soft fiber-constrained linear pneumatic actuators and a rigid rotational housing to offer a comprehensive haptic experience.^360^ This device, made from silicone for compliant touch and rigid precise control, aims to enhance communication and convey tactile information in various applications, freeing the user’s hands for other tasks. Moreover, researchers are developing new actuation mechanisms that leverage significant volumetric changes without the need for a pneumatic source. Miriyev et al.^60^ demonstrated an electrically driven actuator that undergoes large volumetric change at low voltages. This device is a single self-contained soft composite material that combines a silicone elastomer matrix with ethanol, undergoing a liquid–vapor transition through local heating, which causes volume change. It offers the potential for creating haptic devices that do not rely on heavy external sources, providing a high actuation strain from a low-voltage source, which can be practical for untethered devices.

### 6.3 Locomotion of Soft Robotics

In the field of soft robotics, diverse locomotion strategies have been developed to address various real-world applications. The key to these advancements is the use of soft materials, whose inherent flexibility and adaptability enable a wide range of motion capabilities. For instance, aerial soft robots, inspired by avian and insect flight,^361^ demonstrate maneuverability in the air, aquatic soft robots emulate the fluid dynamics of marine organisms to achieve swimming,^362^ and terrestrial robots, including walkers and crawlers, exhibit remarkable adaptability to varied and complex terrains.^4,294^ The following subsection presents the representative soft robots within each motion type, demonstrating the contribution of soft materials to achieving the motion. Figure 8 presents an example of each locomotion type for each movement mechanism.

**Figure 8:**
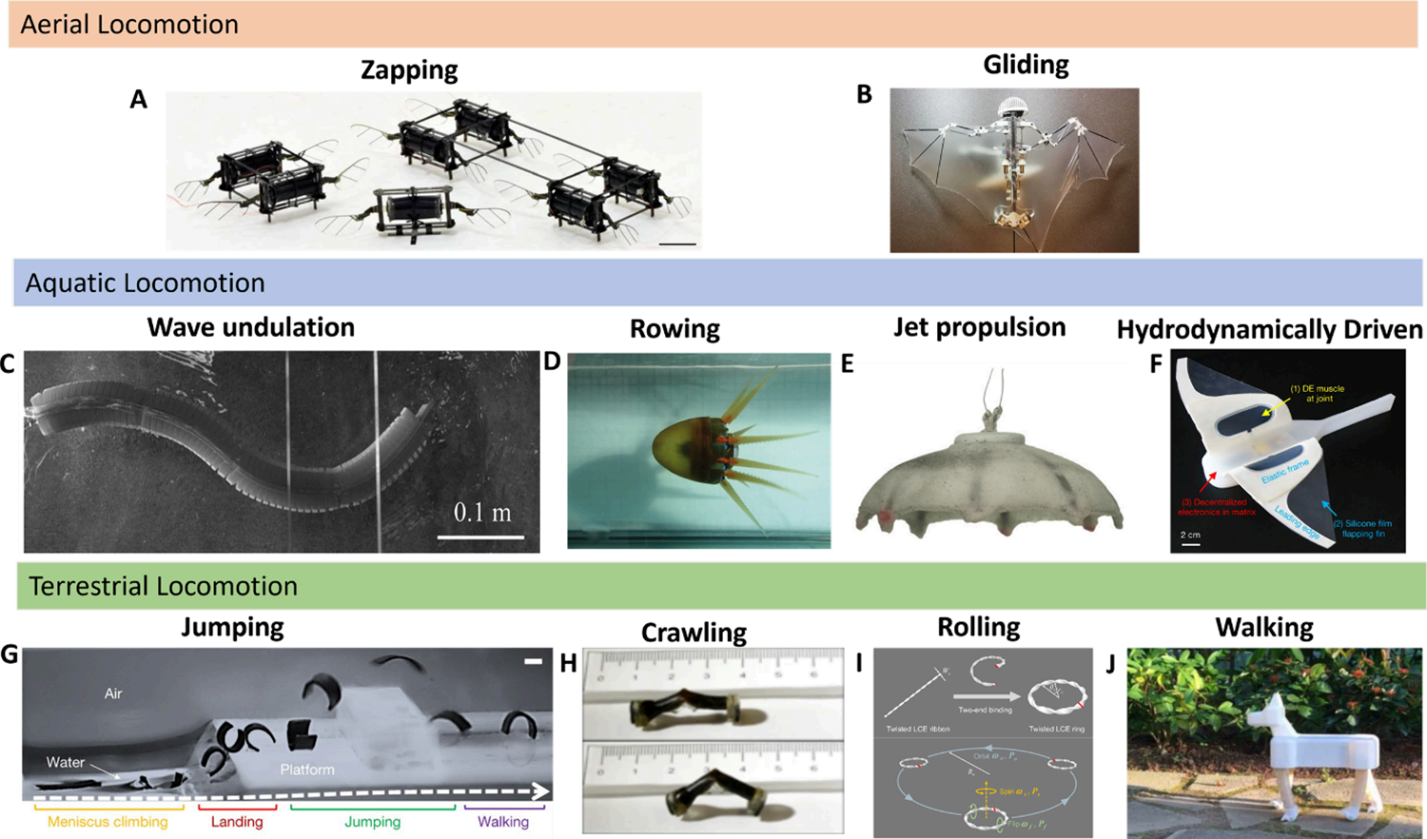
Overview of soft robotics locomotion types, including aerial locomotion. (A) Zapping. Reproduced with permission from ref 365. Copyright 2019 Springer Nature, under Creative Commons Attribution 4.0 International License 4.0 (CC BY). (B) Gliding. Reproduced with permission from ref 369. Copyright 2017 American Association for the Advancement of Science. Aquatic locomotion. (C) Wave undulation. Reproduced with permission from ref 378. Copyright 2018 American Association for the Advancement of Science. (D) Rowing. Reproduced with permission from ref 379. Copyright 2023 Springer Nature, under Creative Commons Attribution 4.0 International License 4.0 (CC BY). (E) Jet Propulsion, Reproduced with permission from ref 380. Copyright 2018 Elsevier. (F) Hydrodynamically Driven. Reproduced with permission from ref 381. Copyright 2021 Springer Nature, under Creative Commons Attribution 4.0 International License 4.0 (CC BY). Terrestrial. (G) Jumping. Reproduced with permission from ref 385. Copyright 2018 Springer Nature under Creative Commons Attribution 4.0 International License 4.0 (CC BY). (H) Crawling. Reproduced with permission from ref 387. Copyright 2019 IEEE. (I) Rolling. Reproduced with permission from ref 391. Copyright 2024 PNAS, under Creative Commons Attribution 4.0 International License 4.0 (CC BY). (J) Walking. Reproduced with permission from ref 395. Copyright 2024 MDPI, under Creative Commons Attribution 4.0 International License 4.0 (CC BY).

#### 6.3.1 Aerial Soft Robots

Aerial soft robots represent an innovative class of flying robots that incorporate flexible and deformable structures, leveraging the compliance and morphing capabilities of soft materials to achieve flight advantages over conventional rigid-bodied aerial robots. These robots are designed to mimic bioinspired flight mechanisms, such as the zapping motion of insect wings,^361,363–365^ the takeoff and gliding dynamics of birds,^361,364,366^ and strong maneuverability similar to that of bees or flies,^365^ and which are all nearly impossible to achieve for rigid bodies.^367^

The most notable work in the field has been done by Chen and Whitney et al.,^365,367^ which has presented an aerial soft zapping robot powered by soft artificial multilayered DEA (Figure 8A). Their research demonstrated both open-loop ascending flight and closed-loop hovering flight in cluttered environments. Based on this foundational work, other researchers have further developed this concept by modifying the DEA actuators for better zapping performance,^368^ refining the soft-robot with both aquatic-aerial movement^361^ and more.^363,364,366^ Despite it was not being made by additive manufacturing, Ramezani et al., who introduced another flight mechanism for soft robots, using a bat-inspired soft robot with silicone membrane wings capable of low-frequency zapping, primarily functioning as a glider^369^ (Figure 8B). The advancements in aerial soft robotics are fundamentally enabled by soft materials, highlighting the novel and complex nature of this field. Despite significant progress, much development is still needed to fully realize the potential of aerial soft robots, particularly in achieving untethered robots with long flight times and more controlled and versatile flight dynamics.

#### 6.3.2 Aquatic Soft Robots

Aquatic soft robots are advanced robotic systems designed to mimic the locomotion of underwater organisms, using soft materials to navigate and operate efficiently in aquatic environments. These robots benefit greatly from soft materials, which provide enhanced flexibility, adaptability, and the ability to withstand the fluid dynamics of underwater settings, allowing for smoother and more natural movements compared to rigid robots.^370–372^ The primary locomotion types in aquatic soft robots include hydrodynamically driven swimming, rowing, wave undulation, and jet propulsion.^362,373^ It should be noted that there are several works on aquatic soft robots crawlers and walkers.^374–376^ This type of application robot will be discussed in the terrestrial soft robot chapter.

##### Wave Undulation

This type of swimming involves generating a traveling wave that propagates from the head to the tail of the fish,^377^ without relying on appendages, through alternating muscle contractions, similar to motions of sea-snakes and eels. Christianson et al. developed a translucent soft robot inspired by eel larvae^378^ (Figure 8C), utilizing frameless fluid electrode DEA. The robot features transparent bimorph actuator segments made from dielectric elastomer and fluid electrodes, allowing for undulatory swimming. The design achieves a maximum swimming speed of 1.9 mm/s and a Froude efficiency of 52%. The actuator’s transparency, with 94% transmittance across the visible spectrum, enables camouflage and optical communication.

##### Rowing

Rowing is a form of drag-based swimming where the appendages, like fins or limbs, push against the water to generate backward thrust. Creatures like octopuses, sea turtles, frogs, and jellyfish move by this method by generating power stocks with flexible soft appendages. Sfakiotakis et al. developed an octopus-inspired multiarm robotic swimmer using polyurethane (PMC-746) for its compliant arms, demonstrating significant flexibility and efficiency in underwater propulsion^379^ (Figure 8D). The robot mimics the octopus’s sculling motion, achieving speeds of up to 0.26 body lengths per second and demonstrating the capability for complex maneuvers and object manipulation.

##### Jet Propulsion

A mode of locomotion is generally used by cephalopods like squids and cuttlefish, where they take a volume of water into a cavity in the body and forcefully expel this water to create jets that generate a thrust to propel themselves forward. Villanueva et al. presented a jellyfish inspired soft robot that moves by contracting its bell to expel water followed by relaxing to allow water to refill the bell, thus creating a cyclical jet that propels it forward. The robot was constructed using a soft silicone matrix bell embedded with SMA composite actuators and achieving neutral buoyancy with extruded polystyrene foam (Figure 8E).^380^

##### Hydrodynamically Driven Swimming (HDS)

The most common mode of locomotion for robotic fish is fins, which generate thrust through their interaction with water. Divided into two propulsion types based on the location of the fins that generate the movement: body and caudal fin (BCF) and median and paired fin (MPF) propulsion. For instance, an untethered snail-fish-like BCF soft robot employs DEA as artificial muscles that move its pectoral silicone soft fins in a flapping motion. The researchers showed that the use of soft materials in constructing the robot enabled it to operate successfully at depths of up to 10,000 m,^381^ as shown in Figure 8F. Another work of Long et al. showed a MPF type tetrapod turtle-like soft robot. They investigated the effect of using four flippers versus two flippers for propulsion. The main conclusion is that while four flippers provide higher accelerations for surge maneuvers, two flippers are more efficient for steady cruising in aquatic tetrapod swimmers.^382^ Additionally, Tan and Cappelleri developed a helical adaptive multimaterial microrobot fabricated using TPP, that swims via BCF like helical propulsion, enabled by a magnetic head and a responsive hydrogel tail. The microrobot demonstrates adaptive swimming in both water and isopropyl alcohol, reaching up to 8.1 body lengths per second.^383^

Despite these advancements, challenges remain in the field of aquatic soft robotics. One significant challenge is achieving smooth vertical movement, as most robots are optimized for horizontal navigation.^362^ Energy efficiency and the stability and durability of soft materials in harsh and dynamic underwater environments also pose significant hurdles. Additionally, the control mechanisms for these robots, especially those using jet propulsion, require further refinement to improve accuracy and reliability. Continued research and development on locomotion mechanisms and materials science are crucial to overcome these challenges and fully realize the potential of aquatic soft robots in various applications.

#### 6.3.3 Terrestrial Soft Robots

Terrestrial locomotion for soft robots involves the navigation across various unstructured terrains and obstacles. Unlike rigid robots, soft robots have the advantage of higher degrees of freedom and material adaptability, allowing them to generate time-varying shapes for an effective interaction with the ground. The main challenge is to control the frictional and reaction forces from the ground to ensure stable and efficient movement.^384^ One of the key difficulties is designing soft robots that are both soft enough to adapt to their environment and rigid enough to exert the necessary force for locomotion. The trade-off between softness for adaptability and rigidity for force exertion is crucial for effective terrestrial locomotion. There are several types of terrestrial locomotion exhibited by soft robots, which include walking, crawling, rolling, and jumping.^4^ For each type, researchers have developed various strategies, as demonstrated in Figure 8, including hybrid designs that combine soft and rigid components to achieve the desired balance for the motion.

##### Jumping

Jumping enables robots to overcome large obstacles by storing and rapidly releasing elastic energy. For instance, Hu et al. developed a small-scale, magneto-elastic soft robot made of silicone elastomer with embedded neodymium–iron–boron microparticles, enabling multimodal locomotion.^385^ The robot’s jumping motion is achieved by using a time-varying magnetic field to control its rigid-body rotation and elastic deformation, allowing it to achieve jumps over obstacles up to several times its own height (Figure 8G). For larger soft robots, it is harder to make a jump due to the increased demands on structural integrity and power required to generate sufficient force to overcome their greater mass. Still, Jeon and Park developed a centimeter sized soft jumping robot that achieves its jumps by utilizing a pneumatic drive system combined with magnetic yield points.^386^ The robot’s structure is composed of EcoFlex 00-50 and Dragon Skin FX Pro silicone polymers, which construct a flexible and durable body. Inside the robot, an air chamber inflates to store energy, while permanent magnets control the expansion. When a certain pressure threshold is reached, the magnetic force is overcome, releasing the stored energy and propelling the robot into the air. This mechanism, along with soft morphing techniques, enables the robot to perform high jumps up to 40 cm (80% its own size).

##### Crawling

Crawling involves body deformations to generate propulsion through frictional forces from the ground. For example, Keneth et al. developed a worm-like soft actuator using 3D printed flexible polyurethane tubes filled with ferrofluid^387,388^ (Figure 8H). The worm actuator moves by utilizing an external magnetic field to contract and relax, achieving forward locomotion through the interaction between the ferrofluid and the magnetic field. Footpads with asymmetric friction were added to the tube’s ends, enabling the worm to move in one direction as the magnetic field alternates, demonstrating the potential for untethered, magnetically controlled soft robotic movement. Another notable work, although it was not made by AM, was created by Shepherd et al., in which they developed a quadrupedal multigait soft robot. The soft robot was fabricated using silicone elastomers (Ecoflex 00-30, Ecoflex 00-50) for the actuating layer and Sylgard 184 for the strain-limiting layer.^389^ The robot achieves walking through the use of pneumatic networks embedded within its elastomeric structure. When these networks are inflated, they cause the legs to bend and extend, producing a coordinated crawling motion.

##### Rolling

Rolling is a unique method of locomotion for soft robots that involves continuous rotation or flipping movements, enabling them to move across surfaces by rolling their entire body. This form of movement can be particularly effective for navigating smooth or slightly uneven terrains, where traditional walking or crawling might be less efficient. Wang et al. developed a triangular closed-chain soft rolling robot using only three curl pneumatic artificial muscles (CPAMs).^390^ The locomotion is achieved through a coordinated sequence of deformations and contraction with the three CPAMs. The robot deforms by bending its edges inward, creating an asymmetry that shifts its mass center out of the supporting points and causes it to roll forward. However, although they achieve fast movement, the robot movement is not continuous, thus limiting its smooth movement. In contrast, in the work by Qi et al., an autonomous soft robot in the form of a defected twisted ring topology composed of LCE was made. The soft robot achieved periodic continuous spin–orbiting motions, as shown in Figure 8I, rolling. The robot’s movements, driven by thermal or photothermal stimuli, involve inside-out flipping, self-spinning around the ring center, and self-orbiting along a circular path, all enabled by the unique twisted ring structure and the defect-induced force asymmetry.^391^

##### Walking

Walking is a locomotion method that allows soft robots to traverse uneven terrain using legged movements. Drotman et al. 3D printed a quadruped soft robot capable of navigating unstructured terrain such as large rocks, sand pebbles, and slippery valley.^392^ The soft robot leg actuators were printed from a combination of a rubbery soft material (TangoBlackPlus) and a rigid material (VeroClear), balancing the compliance for interaction with varying environments and the stiffness for load-bearing. Matia et al. developed a soft robotic actuator composed of SLA 3D printed elastomer bellows connected by fluidic tubes, using viscous-driven pressure gradients to achieve complex motion from a single input.^393^ Embedded in a six-legged soft robot, these actuators enabled untethered walking at 0.05 body lengths/s, demonstrating how morphology-based control can produce rich locomotion without electronic feedback. In another work, Tang et al. presented a versatile design for high-performance walking soft robots by bistable mechanisms inspired by the spine movements of fast terrestrial animals, such as cheetahs.^394^ The soft robots were constructed by silicone elastomers for the pneumatic actuators and spring steel, that is, act as tendons for energy storage for the bistable mechanisms. The pneumatic actuation is bending the tendon, which then releases the contract and creates the movement. A recent report by Le et al. demonstrated an untethered soft robotic dog capable of standing and fast trotting utilizing jointless and resilient soft legs made entirely of silicone rubber. The robot employs precharged pneumatic actuators for its legs, allowing it to achieve a trotting speed of up to 23 cm/s (0.97 body lengths per second) and navigate various terrains^395^ (Figure 8J).

Terrestrial locomotion in soft robots has advanced significantly, but several challenges remain. One major issue is the need for higher speeds and more robust control mechanisms. Current walking robots are slower compared with biological counterparts, and coupling effects in leg trajectories need to be minimized. Crawling robots face energy losses due to frictional slips, and rolling robots struggle with control at high speeds due to gaps in their wheel-like shapes. Additionally, precise control of jumping trajectories and the development of effective climbing locomotion are areas needing further research.^4,294^ Advancements in materials with lower viscoelasticity, better actuation methods, and improved sensor integration will be crucial for overcoming these challenges and enhancing the performance of terrestrial locomotion in soft robots.

### 6.4 Soft Grippers and Manipulators

Soft robotic grippers, one of the most extensively studied fields in soft robotics, are fabricated with flexible soft materials to achieve compliance to perform gripping and manipulation without failure. The key advantage of soft grippers lies in their ability to conform to the objects they grasp, reducing the risk of damage and enhancing the interaction between the robot and its environment.^396^ This unique capability is particularly beneficial in fields such as medical surgery, where precision and safety are necessary, in agriculture, where delicate produce must be handled with care.^397^

Soft robotic grippers come in various forms and are designed to mimic different natural and artificial gripping mechanisms. These include finger-like, tentacle, and universal grippers.^396^ Finger-like grippers are designed to mimic human kinesiology, typically with multiple fingers that can wrap around objects. Each finger may be independently controlled or work simultaneously, while each finger may be independently controlled or actuated simultaneously, with most designs offering 2–3 degrees of freedom (DOFs) per finger.^396^ Tentacle-like grippers are inspired by octopus arms^398–401^ or elephant trunks^204^ and are characterized by their continuous and highly flexible structure, which allows for smooth bending and twisting motions.^396^ These grippers can wrap around objects of various shapes and sizes. They are usually made of highly flexible materials that allow for extensive bending and twisting motions. Tentacle-like grippers are particularly useful for handling objects in cluttered or confined spaces where traditional grippers might struggle.^401^ The key distinction between finger and tentacle-like grippers lies in their mechanics: finger-like grippers rely on discrete segments and joints, whereas tentacle grippers achieve motion through continuum deformation and do not exhibit clear joint boundaries. Universal grippers are those, which do not have a confined shape, are designed to conform and fix their shape to the desired object.^402^ Each type of gripper design offers its advantages and is suited to different applications. Additionally, to achieve the actuation of the gripper several mechanisms have been studied and classified as follows: pneumatic, cable-driven, electroactive polymers, shape memory actuation, and jamming.^396,397,403^ Figure 9 shows representative grippers for each actuation mechanism for different designs. In this section, a short overview of each of these actuation mechanisms will be briefly presented, including their most representative work highlighting their potential of application.

**Figure 9:**
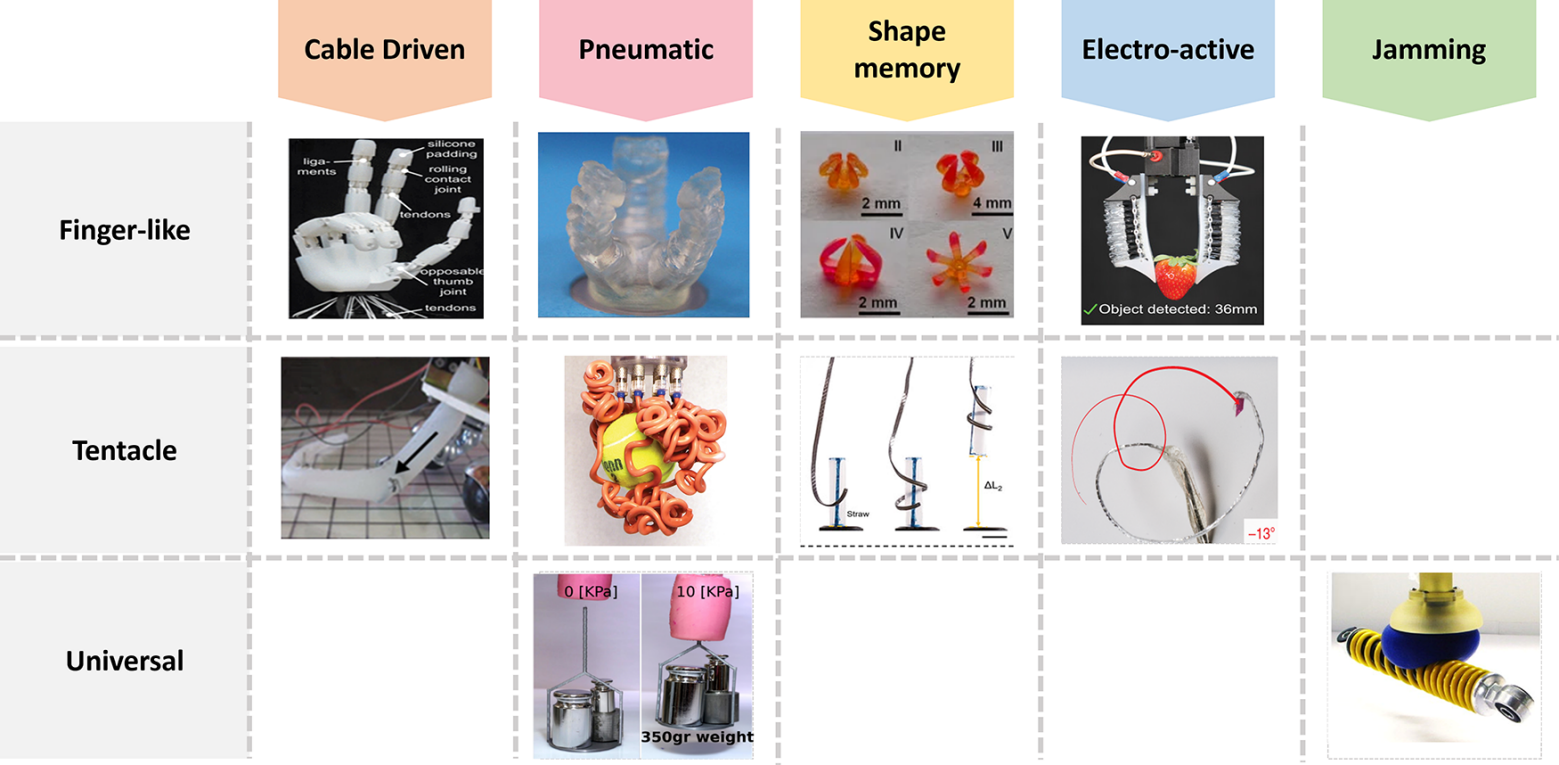
Classification of soft robotic grippers by actuation mechanism and gripper type. The table categorizes soft robotic grippers into five actuation mechanisms: Cable-driven, pneumatic, electroactive, shape-morphing, and jamming. Each mechanism is further divided into three gripper types: finger-like, tentacle, and universal. The cable-driven section includes a tendon driven fingers. Reproduced with permission from ref 407. Copyright 2020 Frontiers, under Creative Commons Attribution 4.0 International License 4.0 (CC BY). Cable-driven tentacle gripper. Reproduced with permission from ref 408. Copyright 2019 Springer Nature, under Creative Commons Attribution 4.0 International License 4.0 (CC BY). The pneumatic section includes a schematic of a pneumatically 3D printed stretchable gripper. Reproduced with permission from ref 32. Copyright 2019 Wiley-VCH, under Creative Commons Attribution 4.0 International License 4.0 (CC BY). Pneumatic tentacle based on active entanglement. Reproduced with permission from ref 401. Copyright 2022, PNAS, Creative Commons Attribution-Non Commercial-No Derivatives License 4.0 (CC BY-NC-ND). Pneumatic universal compliant gripper. Reproduced with permission from ref 75. Copyright 2023 Royal Chemical Society. Shape memory section includes: a schematic of 4D printed multimaterial shape memory polymer (SMP) gripper. Reproduced with permission from ref 413. Copyright 2019 Springer Nature, under Creative Commons Attribution 4.0 International License 4.0 (CC BY). Tentacle-like soft hydraulic actuator based on SMP. Reproduced with permission from ref 414. Copyright 2024 Wiley-VCH, under Creative Commons Attribution 4.0 International License 4.0 (CC BY). Electroactive section includes finger-like gripper based on HASEL actuators. Reproduced with permission from ref 410. Copyright 2020 Wiley-VCH, under Creative Commons Attribution 4.0 International License 4.0 (CC BY). Tendril-like soft gripper based on reversible osmotic actuation. Reproduced with permission from ref 412. Copyright 2019 Springer Nature, under Creative Commons Attribution 4.0 International License 4.0 (CC BY). The jamming section includes universal granular-based jamming gripper. Reproduced with permission from ref 402. Copyright 2010 published by PNAS.

#### 6.4.1 Pneumatic Actuation

Pneumatic actuators utilize air pressure to actuate movement and typically consist of networks of air channels within soft materials that inflate to create bending and gripping motions. The most common example of pneumatic devices is the McKibbin artificial muscle from which many soft robot manipulators and grippers are constructed. However, despite their simple mechanism, these devices often lack sufficient compliance for delicate grasping and can be complex to fabricate.^79,404^ In contrast, simple soft pneumatic grippers made of silicone rubber can be easily fabricated using molding techniques,^405^ and they are widely applied in the industry.^406^ Despite their simplicity, these methods are limited in design possibilities. Recent advancements in materials science have enabled the 3D printing of pneumatic grippers using stretchable and compliant materials, vastly expanding the design possibilities. For instance, Patel et al. developed highly stretchable and UV-curable polyurethane suitable for fabrication of soft robotic grippers^32^ (Figure 9, pneumatic, finger-like). However, these materials are characterized by a lack of compressibility that results in failure to grip various structures effectively. Further development in this area has shown that by embedding micropores within the stretchable matrix, the material gains compressibility, allowing it to conform to any object and successfully grasp various objects without failure.^204^ In addition, with the same material, Bliah et al.^75^ has demonstrated a universal design for a pneumatic gripper consisting on a monolithic design with radial chambers and an elastic lattice layer to achieve effective grasping through force closure and compliant interaction. The gripper operates by expanding its internal walls upon positive pressure application, enabling secure and adaptable object manipulation. Demonstrating its capabilities, it could grasp a 23G needle (0.64 mm diameter) and lift objects up to 12 times its weight (Figure 9, pneumatic, universal structure).

#### 6.4.2 Cable-Driven Actuation

Cable-driven grippers operate by using tendons or cables that mimic the function of biological tendons. These grippers can achieve precise movements and are often used in applications requiring high dexterity. For example, Kim and Cha developed a soft pneumatic gripper utilizing a novel tendon-driven soft origami pump.^407^ The gripper comprises three pneumatic soft actuators made of Ecoflex 00-30 silicone, controlled by an origami pump fabricated from a Kresling-patterned polypropylene film. This design eliminates the need for an external air compressor, allowing for a compact and efficient system. The gripper demonstrated effective grasping capabilities with various objects, showcasing its potential for diverse applications in soft robotics (Figure 9, Cable-driven, finger-like structure). In general, the cable driven grippers hand has a limited degree of freedom due to constraint by the joints. A study that potentially overcomes this issue has been presented by Lee et al., who developed a soft robotic gripper with continuous fingers using long SMA tendons embedded in a PDMS matrix408 (Figure 9, Cable-driven, tentacle design). The SMA tendons, made of Flexinol LT wires, are free-sliding within silicone rubber tubes, allowing for large bending deformations independent of the matrix length. The gripper achieves bending angles up to 400° and a tip force of 0.89 N. The modular design, featuring a tendon-driven mechanism with V-shaped bearings, demonstrated the ability to grasp various objects weighing up to.^1,5^ kg. Another bioinspired cable driven tentacle has been presented by Calisti et al., which demonstrated a tendon-driven octopus-inspired arm.^399^ The arm replicates the octopus’s ability to elongate, shorten, and bend. This bioinspired design allows the robot to achieve pushing-based locomotion with a high degree of freedom and object grasping with minimal control.

#### 6.4.3 Electroactive Actuation

Electroactive polymer grippers use materials that deform in response to an electric field. Dielectric elastomers are a common choice, providing rapid response times and significant actuation strains. As for the pneumatic system, the artificial muscle based on electroactive polymer is the hydraulically amplified self-healing electrostatic (HASEL) actuators.^409^ Yoder et al. developed a versatile gripper utilizing HASEL actuator, which combines soft and electrically driven components with capacitive self-sensing for real-time pick verification and object size detection.^410^ The gripper was constructed from multimaterial actuators made of Mylar film pouches filled with silicone oil, which bend and grip objects when high voltage is applied. The integrated high-voltage driving electronics enable rapid and precise control, allowing the gripper to perform various gripping tasks with high speed and low power consumption (Figure 9, Electroactive polymers, finger-like structure). Another pioneering work in the field was done by Shintake et al., which introduced a versatile soft gripper that uses intrinsic electroadhesion based on multifunctional electroactive polymer actuators.^411^ These grippers utilize DEAs made from a prestretched elastomer membrane (Nusil CF19-2186) with patterned compliant electrodes laminated between two passive silicone (Sylgard 184) layers. The unique electrode configuration maximizes both electroadhesion and electrostatic actuation, allowing the gripper to handle a wide range of objects, from fragile items such as a raw chicken egg to flat sheets of paper. Additionally, Must et al.^412^ have developed a variable-stiffness tendril-like soft robot based on reversible osmotic actuation (Figure 9, electroactive polymers, tentacle design). This design, inspired by plant movements, employs electrosorption of ions on flexible porous carbon electrodes to achieve reversible stiffening and actuation at safe, low voltages, resulting in a tendril-like structure that can bend and rotate.

#### 6.4.4 Shape Morphing-Based Actuation

These grippers leverage materials that can change their shape when exposed to external stimuli such as SMP, SMA, and LCE. These grippers are useful in compact applications where grasping fixation is needed. For instance, Ge et al.’s fabricated multimaterial SMP architectures by 4D printing using high-resolution PμSL.^413^ They developed a photocurable methacrylate-based copolymer with tailorable thermomechanical properties capable of forming complex 3D structures that can transform their shape in response to heat. They demonstrated a material with a thermos-responsive gripper capable of closing and fixating on various objects (Figure 9, shape morphing, finger-like structure). While making simple open and closing movements with shape memory polymers is straightforward, preparing polymers to act as tentacles that can wrap around objects is quite challenging. Qing et al. demonstrated this concept by developing fully 3D-printed miniature soft hydraulic SMP actuators for morphing and manipulation.^414^ Utilizing a combination of stiff SMPs and soft elastomers, these actuators can wrap around and securely grasp objects (Figure 9, shape morphing, tentacle design), achieving fast, versatile shape morphing and locking for noninvasive manipulation and energy-efficient applications. Finally, Hsu et al. developed a four-arm soft microgripper via two-photon polymerization using a liquid crystalline elastomer (LCE) formulation based on RM82, RM257, and the E7 liquid crystal mixture. The gripper, with an arm length of ∼50 μm, was functionalized postprinting with photoresponsive dyes, enabling reversible shape changes under visible light with response times as fast as 35 ms and programmable actuation using multiple wavelengths.^415^

#### 6.4.5 Jamming Grippers

Such grippers use granular materials encased in a flexible membrane, which can transition between fluid-like and solidlike states under vacuum. This enables the gripper to conform to the shape of the object and then harden to secure the grip. These grippers often use materials like coffee grounds or ground-up rubber encased in a silicone membrane that can be fabricated by traditional methods or by AM for more complex structures.^416^ An example of their application is in robotic pick-and-place tasks where objects vary widely in shape and size. Jamming grippers are effective for securely holding irregularly shaped items without damaging them.^402^

## 7 Challenges and Future Perspectives

Despite significant advancements during the past years in the field of 3D printed soft robotics, several challenges still persist that hinder the full realization of their potential. Addressing these challenges is crucial for the widespread adoption and application of soft robots in various fields. This section discusses the remaining challenges from our point of view and explores the areas where future development is required.

### 7.1 Additive Manufacturing vs Conventional Fabrication Methods

As discussed throughout this review, AM has significantly advanced the field of soft robotics by enabling the creation of complex geometries, integrated multimaterial systems, and spatially tuned mechanical properties. However, AM is not universally superior to conventional methods. For instance, kirigami- and origami-based structures (section 5.3) still rely on precise planar fabrication and folding steps more easily achieved by laser cutting than by layer-by-layer printing. Moreover, many advanced soft robots, especially untethered systems, require the integration of discrete components, such as batteries, microcontrollers, valves, and fluidic or magnetic subsystems. Until now, these components cannot yet be monolithically printed, necessitating postprint assembly using conventional manufacturing or hybrid techniques. This reliance on external modules presents challenges not only for seamless integration but also for reliability, compactness, and biocompatibility, particularly in miniaturized or implantable devices. In such cases, AM’s benefit of monolithic design is diminished, and traditional assembly may offer superior control over component placement and function. Furthermore, the limited availability of printable conductive and magnetic materials restricts the creation of fully autonomous, multifunctional soft robots, making it difficult to integrate actuation, sensing, and control within a single fabrication process. Finally, AM remains more suitable for prototyping and custom fabrication than for mass manufacturing, where molding and casting remain dominant due to their lower cost, faster throughput, and compatibility with established materials. However, fabricating very complex structures is either impossible to make by conventional processes or requires very costly instrumentation (molds) or a postassembly process. Overcoming the above limitations will require progress in hybrid fabrication, modular system design, and the development of new AM-compatible functional materials.

### 7.2 Materials Development

One of the primary challenges in the fabrication of soft robots lies in the development of suitable materials. The ideal material must exhibit properties such as compliance, flexibility, and biocompatibility while being compatible with the fabrication technique. Current materials often face trade-offs between mechanical strength and elasticity, limiting their application in diverse environments. Moreover, ensuring the reliability and durability of soft robots in various operational modes is essential. Factors such as material fatigue, environmental degradation, and mechanical wear can significantly reduce the operational lifespan of soft robots. Enhancing material resilience through advanced formulations and incorporating self-healing and self-cleaning properties can address these issues. In particular, material systems such as organogels, high-viscosity functional composites, and biocompatible elastomers such as PDMS remain underdeveloped for AM, requiring new formulations and process-compatible chemistries to unlock their full potential in soft robotics. Future research should focus on developing such novel materials that can maintain these requirements while being suitable for advanced fabrication techniques such as 3D printing.

### 7.3 Multimaterial 3D Printing

Multimaterial 3D printing offers the potential to create complex structures with varied mechanical properties, yet it poses its own set of challenges. Ensuring proper adhesion between different materials, maintaining precision in the deposition process, and preventing cross-contamination are critical issues that need to be addressed. In recent years, there have been some publications in multimaterials printing of soft robotics, mainly using silicones by extrusion-type technologies and using several nozzles. A significant gap in research is the lack of studies on multiple wavelength printing, which could facilitate more efficient multimaterial integration at a higher resolution than DIW technologies. Advancements in this field will help improve actuation and sensing mechanisms and enable new designs that were not possible using a single material. Multimaterial printing at high resolution can lead fabricating functional soft robots that include sensing, feedback, and actuation in a single printing process. This will obviously require the development of new materials, such as in multiwavelength stereolithography processes that require two different photopolymerization mechanisms.

### 7.4 Design and Modeling

Developing new 3D printing materials would enable a higher degree of freedom of design while tailoring the mechanical and physicochemical properties of the printed objects. Having a wide library of printable materials, for example, with controllable mechanical properties by interpenetrating networks, will enable formation of soft robots with gradual mechanical stifness. It is expected that advanced computational modeling techniques including AI will enable new opportunities in design and robotics behavior prediction according to material’s properties.

### 7.5 Integration of Sensors

Seamless integration of sensors within actuators of soft robots still presents significant barriers. Traditional rigid sensors are often incompatible with the compliant nature of the soft robots, leading to integration challenges, often by post fabrication of individual components. Advances in flexible electronics, optics, and soft sensors that can be embedded directly within the robot during the printing process are crucial for overcoming the post integration process. A key development area is the creation of multipurpose sensors capable of performing various functions within a single unit. Currently, sensors are typically designed for single purposes, such as contact or slip detection, which complicates the manufacturing process and increases the complexity of the robotic systems due to the need to place and connect different sensors in one device. Moreover, we envision that sensing can be achieved while the material functions as both actuator and sensor. This dual functionality will simplify the design and fabrication and will improve the overall performance of the soft robotic device.

### 7.6 Control and Feedback Platforms

The development of dedicated controllers and stable interfaces for soft robots is another area that requires significant attention. The control is often achieved by using electrical signals and, to a lesser extent, optical signals. From a materials point of view, embedded flexible electrical connectors and optical fibers would enable signal processing and reliability of the printed device communications between the different components and the external environment. In addition, the dynamic and flexible nature of soft robots makes it difficult to apply conventional control algorithms effectively for materials with nonlinear behavior and unpredictable deformations. Research into machine learning, AI, and adaptive control strategies could provide robust solutions for these challenges.
